# Effects of dietary fiber on the composition, function, and symbiotic interactions of intestinal microbiota in pre-weaned calves

**DOI:** 10.3389/fmicb.2025.1554484

**Published:** 2025-03-25

**Authors:** Wentao Lu, Xia Yi, Yuhan Ge, Xinyue Zhang, Kaidi Shen, Haohua Zhuang, Zhaoju Deng, Dengke Liu, Jie Cao, Chong Ma

**Affiliations:** ^1^College of Veterinary Medicine, China Agricultural University, Beijing, China; ^2^School of Chemistry, University of Bristol, Bristol, United Kingdom; ^3^Hebei Shounong Modern Agricultural Technology Co., LTD., Dingzhou, China

**Keywords:** calves, dietary fiber, gastrointestinal health, *Bifidobacterium*, *Prevotella*, microbial interactions, intestinal homeostasis

## Abstract

**Introduction:**

Dietary fiber plays a crucial role in maintaining gastrointestinal health. However, its protective effects on the intestinal health of calves remain to be fully elucidated. This study aimed to investigate the impact of dietary fiber supplementation on the intestinal microbiota of pre-weaned calves and its potential role in modulating microbial metabolic pathways.

**Methods:**

A randomized controlled trial was conducted, enrolling 135 calves that were randomly assigned into three groups: (1) inulin supplementation, (2) psyllium husk powder (PHP) supplementation, and (3) a control group receiving no dietary fiber. Fecal microbiota samples were collected from calves without diarrhea at five time points (0, 7, 14, 28, and 56 days of age). Metagenomic sequencing was performed to analyze microbial composition and functional pathways. Additionally, a differential analysis of carbohydrate-active enzymes (CAZymes) was performed to evaluate the effect of dietary fiber on carbohydrate metabolism enzyme activity within the intestinal microbiota.

**Results:**

Calves supplemented with dietary fiber exhibited a significant increase in the abundance of *Bifidobacterium* and *Prevotella* compared to the control group. These bacterial genera contributed to intestinal protection by modulating secondary bile acid metabolism and flavonoid metabolism pathways. CAZymes differential analysis revealed an increased abundance of carbohydrate metabolism enzymes in response to dietary fiber supplementation, with distinct microbial community compositions observed among different fiber treatments. Notably, at 56 days of age, calves fed PHP harbored intergeneric symbiotic clusters comprising *Clostridium, Prevotella*, and *Bacteroides*, suggesting a cooperative microbial network that may contribute to intestinal homeostasis.

**Discussion:**

The findings of this study highlight the beneficial effects of dietary fiber on calf intestinal microbiota, particularly in enhancing microbial diversity and enzymatic activity related to carbohydrate metabolism. The observed microbial symbiosis in PHP-fed calves suggests a potential role in maintaining intestinal homeostasis. These insights provide a theoretical foundation for optimizing dietary interventions to promote gut health in calves during the transition period. Further research is warranted to explore the mechanistic interactions between dietary fiber, gut microbiota, and host health outcomes.

## 1 Introduction

The health of calves is the foundation of a successful dairy farming industry. However, diarrhea remains a serious global challenge, contributing substantially to calf mortality and culling rates. Improving intestinal health helps calves fight off infections, grow stronger, and survive early-life challenges (Malmuthuge and Guan, 2017; Du et al., 2023). Research has demonstrated that calves experiencing diarrhea before weaning often face long-term effects, including decreased reproductive efficiency, reduced 305-day mature equivalent milk production (305ME) during their first lactation, and slower average daily gain (ADG) (Abuelo et al., 2021). According to the United States National Animal Health Monitoring System (NAHMS), nearly one-third of pre-weaned calves (33.8%) experience at least one illness, and over half of these cases (50.9%) are caused by digestive problems, such as diarrhea (NAHMS, 2021). Digestive issues are even more serious in newborn calves, causing about 32% of all deaths during this stage (Cho and Yoon, 2014; Urie et al., 2018). These numbers indicate how critical it is to maintain a healthy digestive system in young calves, especially for their long-term health and reproductivity.

Intestinal health is fundamental to the overall physiological health, with the gut microbiota playing a critical role in both maintaining the structure and function of the intestinal epithelium and the regulating immune responses (Zhou M. et al., 2024). A balanced intestinal environment provides a defense system, preventing harmful microorganisms from entering the bloodstream, maintaining immune balance to avoid overactivity, and strengthening the intestinal barrier through complex interactions among microbial communities, their metabolites, and epithelial cells (Kayama et al., 2020; Yue et al., 2023; Zhou P. et al., 2024). Additionally, the intestinal microbiota occupies ecological niches within the gastrointestinal tract, restricting space and resources available to pathogens, which helps maintain intestinal homeostasis (Dablool et al., 2024; Fauzia et al., 2024). The gut microbiota plays a significant role during early life, as it forms the basis for the development of both intestinal mucosal and systemic immune systems (Rodríguez et al., 2015). This early microbial ecosystem is highly dynamic, undergoing rapid changes that coincide with critical phases of intestinal and immune system maturation, particularly in pre-weaned calves (Hennessy et al., 2020). However, disruptions to the gut microbiota during this formative stage can lead to long-term dysbiosis, which increases susceptibility to chronic health issues (Belkaid and Hand, 2014; Sarkar et al., 2021). Thus, fostering the growth of a healthy intestinal microbiota during this key developmental window in calves is essential for reinforcing the intestinal barrier, maintaining gastrointestinal health, and laying the foundation for their long-term growth and resilience.

Dietary fiber has been widely recognized for its important role in maintaining gastrointestinal health (Elihasridas et al., 2023). A previous study demonstrated the ability of fiber to stimulate gastrointestinal motility, preserve the integrity of the intestinal mucosa, and facilitate the colonization and fermentation of beneficial microbiota, which resulted in the production of short-chain fatty acids (Gill et al., 2021; Jiajun et al., 2024). Furthermore, fiber supplementation has emerged as an effective intervention for mitigating antibiotic-induced dysbiosis by modulating the redox potential of the intestine (Penumutchu et al., 2023). Of the various dietary fibers, inulin has been shown to enhance the abundance of *Bifidobacterium* in the gut microbiota and increase the concentration of fecal short-chain fatty acids (Le Bastard et al., 2020a; Chen et al., 2023). Similarly, psyllium husk has been shown to promote the growth of butyrate-producing bacteria, improve stool consistency through enhanced water absorption, and fortify the intestinal barrier by reducing proinflammatory cytokines IL-1 and IL-6, as well as serum tryptophan levels (Hu et al., 2023; Lai et al., 2023; Garg et al., 2024). These findings underscore the potential of inulin and psyllium husk as functional dietary fibers that can effectively regulate the composition of the gut microbiota, reinforce the intestinal barrier integrity, and optimize stool quality. Thus, both inulin and psyllium husk are promising strategies for enhancing the gastrointestinal health of pre-weaned calves.

Our study aimed to fill this knowledge gap regarding the potential of inulin and psyllium husk as beneficial modulators of the gut microbiota of pre-weaned calves. We performed a feeding trial to assess how inulin and psyllium husk influence the intestinal microbiota composition and function in pre-weaned calves. Fecal samples were collected at multiple time points to track microbial changes and evaluate interactions within the microbial community. These findings provide valuable insights into the mechanisms by which these dietary fibers support gut health and reduce diarrhea risk in pre-weaned calves.

## 2 Materials and methods

### 2.1 Animal and study design

This study was conducted at a dairy farm in Hebei Province, China. A total of 136 healthy newborn Holstein female calves were selected based on health criteria developed by McGuirk and Peek (2014). These criteria evaluate calf health by checking their mental state, nasal and eye discharge, and coughing. All calves received colostrum shortly after birth and were individually housed in pens.

The calves were divided into three groups: the control group (CON), the psyllium husk powder group (PHP), and the inulin group (INU). Details about the group assignments are shown in Table 1. Starting on the second day after birth, PHP group calves received psyllium husk powder, and INU group calves were supplemented with inulin. The control group did not receive any dietary fiber. The feeding schedule and composition of the feed are detailed in Table 1.

**Table 1:** Basic information, feeding scheme, and feed composition of pre-weaning calves.

**Category**	**Item**	**CON group**	**PHP group**	**INU group**	**Remarks**
Basic information	Sample size (*n*)	45	46	45	Number of calves in each group
	Mean parity of cows (times)	2.24 ± 1.76	2.72 ± 2.10	2.00 ± 1.13	*F* = 2.059, *P* = 0.132
	Birth weight (kg)	37.36 ± 4.39	36.89 ± 3.29	37.29 ± 5.15	*F* = 0.153, *P* = 0.858
Feeding scheme	Days 1–7 (milk volume/meal)	3.0 L	3.0 L	3.0 L	Pasteurized milk, 3 meals/day
	Days 8–15 (milk volume/meal)	3.5 L	3.5 L	3.5 L	Pasteurized milk, 3 meals/day
	Days 16–28 (milk volume/meal)	2.5 L	2.5 L	2.5 L	Pasteurized milk, 3 meals/day
	Days 29–49 (milk volume/meal)	2.5 L	2.5 L	2.5 L	Milk replacer, 3 meals/day
	Days 50–52 (milk volume/meal)	2.5 L	2.5 L	2.5 L	Milk replacer, 2 meals/day
	Days 53–56 (milk volume/meal)	2.5 L	2.5 L	2.5 L	Milk replacer, 2 meals/day
Dietary fiber	Days 2–28 (g/day)	None	10	10	Supplemented with psyllium husk or inulin
	Days 29–56 (g/day)	None	20	20	
Feed composition	Crude protein (CP), %	≥20.0	≥20.0	≥20.0	Similar in milk replacer and starter feed
	Crude fat, %	≥16.5	≥16.5	≥16.5	Milk replacer
	Crude ash, %	≤ 10.0	≤ 10.0	≤ 10.0	Milk replacer
	Crude fiber, %	≤ 1.5	≤ 1.5	≤ 1.5	Milk replacer
	Crude fiber (starter feed), %	≤ 8	≤ 8	≤ 8	Starter feed
	Calcium (Ca), %	≥0.5	≥0.5	≥0.5	0.4–1.5% in starter feed
	Phosphorus (P), %	≥0.5	≥0.5	≥0.5	0.4–1.0% in starter feed

Fecal samples were collected on days 0, 7, 14, 28, and 56. These samples were stored at −80°C to maintain their quality for analysis. Fecal consistency scores were recorded daily for each calf throughout the trial. The scores ranged from 0 to 3 and were applied through observing the freshest feces visible on the bedding. A fecal consistency score of 2 or higher indicated “diarrhea” in a calf, while a score below 2 indicated a “healthy” calf. The duration of diarrhea was recorded when a calf exhibited signs of diarrhea, with the onset marked as the age in days when a diarrhea score of 2 was first recorded. The end of a diarrhea episode was identified as the day when the score was below 2. The duration of diarrhea was calculated as the lapse of time between the two dates, representing the occurrence of diarrhea in the calf (McGuirk, 2008). Nine healthy calves from each group that did not develop diarrhea during the study were selected for further analysis. These samples were used to examine how dietary fiber affected the gut microbiota of the calves.

### 2.2 DNA extraction and metagenome sequencing

In this study, complete microbial DNA was extracted from fecal samples using a magnetic soil and fecal DNA extraction kit (DP712, China TianGen Biotechnology Co., Ltd.). Approximately 0.25–0.5 g of each sample was mixed with lysis buffers and grinding beads, followed by mechanical disruption and thermal lysis. After centrifugation, the supernatant was transferred for further purification. DNA was then precipitated using binding buffer and magnetic beads, allowing selective adsorption of DNA. The beads were washed with ethanol-based buffers to remove contaminants, and DNA was finally eluted in Buffer TB. The purified DNA was stored at −20°C for downstream applications. Metagenomic sequencing was conducted on the Illumina NovaSeq 6000 platform, generating paired-end reads of 150 base pairs, by China TianGen Biotechnology Co., Ltd. The sequencing libraries were prepared using the TruSeq DNA Sample Prep Kit, with the 5′ adapter sequence: 5′-AATGATACGGCGACCACCGAGATCTACACTCTTTCCCTACACGACGCTCTTCCGATCT-3′ and the 3′ adapter sequence: 5′-GATCGGAAGAGCACACGTCTGAACTCCAGTCAC(index)ATCTCGTATGCCGTCTTCTGCTTG-3′.

### 2.3 Metagenomic data overview

After sequencing, low-quality reads were removed using Fastp (v0.24.4), yielding a total of 2,779,248,798 reads. Host DNA was removed using Bowtie2 (v2.5.2) within Kneaddata (v0.12.0), resulting in 1,586,617,206 clean reads, with an average of 9,775,668.787 clean reads per sample (58.17% of the total reads) (Supplementary Table S1).

### 2.4 Bioinformatic and statistical analysis

Bioinformatic and statistical analyses were conducted using established methodologies (Liu et al., 2020). The raw data were subjected to quality control using Fastp (v0.23.4), followed by the removal of host sequences derived from NCBI (GenBank assembly ID: GCA_021347905.1, https://www.ncbi.nlm.nih.gov/datasets/genome/GCA_021347905.1/) using Bowtie2 (v2.5.2) within Kneaddata (v0.12.0) to obtain clean data. Subsequently, the clean data were assembled using megaHit (V1.2.9). Following read assembly, species annotation was conducted using Kraken2 (v2.1.3), while KEGG and CAZy database annotations (based on eggNOGDB version: 5.0.2) were performed using Diamond (v2.0.15) (Huang et al., 2018; Zhang et al., 2018; Zheng et al., 2023).

To analyze temporal changes in fecal microbiota composition, we assessed α diversity using the Shannon diversity index and Abundance-based Coverage Estimator (ACE) index. β diversity was evaluated using canonical principal coordinate analysis (CPCoA). Linear regression analysis was performed to assess the relationship between dietary fiber intervention and microbial diversity, with *R*^2^ values calculated for ACE and Shannon indices.

Metagenomic statistical analysis was conducted using R software version 4.3.1 (https://www.R-project.org/). The *P*-values for α diversity indices (ACE and Shannon) were calculated using ANOVA with *post-hoc* Tukey HSD test for multiple comparisons. The β diversity (distance values calculated using Chao) was analyzed with cPCoA, and *P*-values were computed using an ANOVA-like permutation test. For Spearman correlation analysis, *P*-values were not adjusted for multiple testing. Similarly, group comparisons of significantly different taxa and functional features (e.g., KEGG/CAZy) were performed without *P*-value correction. As this study is exploratory, unadjusted *P*-values were used to better reveal potential changes in the abundance and functionality of fecal microbiota in calves.

Visualization of α-diversity, β-diversity, and species stacked bar plots was performed using ggplot2 (Valero-Mora, 2010). The differential analysis of species and KEGG/CAZy database annotation results was performed using STAMP (statistical analysis of taxonomic and functional profiles, version: 2.1.3) and LEfSe (linear discriminant analysis effect size). Simple linear analysis was conducted using GraphPad Prism (v9.5.1), while Spearman correlation was calculated using WGCNA (https://cran.r-project.org/web/packages/WGCNA/index.html, Langfelder and Horvath, 2008). Co-occurrence networks were visualized using Gephi (https://gephi.github.io/, Bastian et al., 2009).

## 3 Results

### 3.1 Longitudinal changes in calf fecal microbiota (day 0–56)

**Figure 1:**
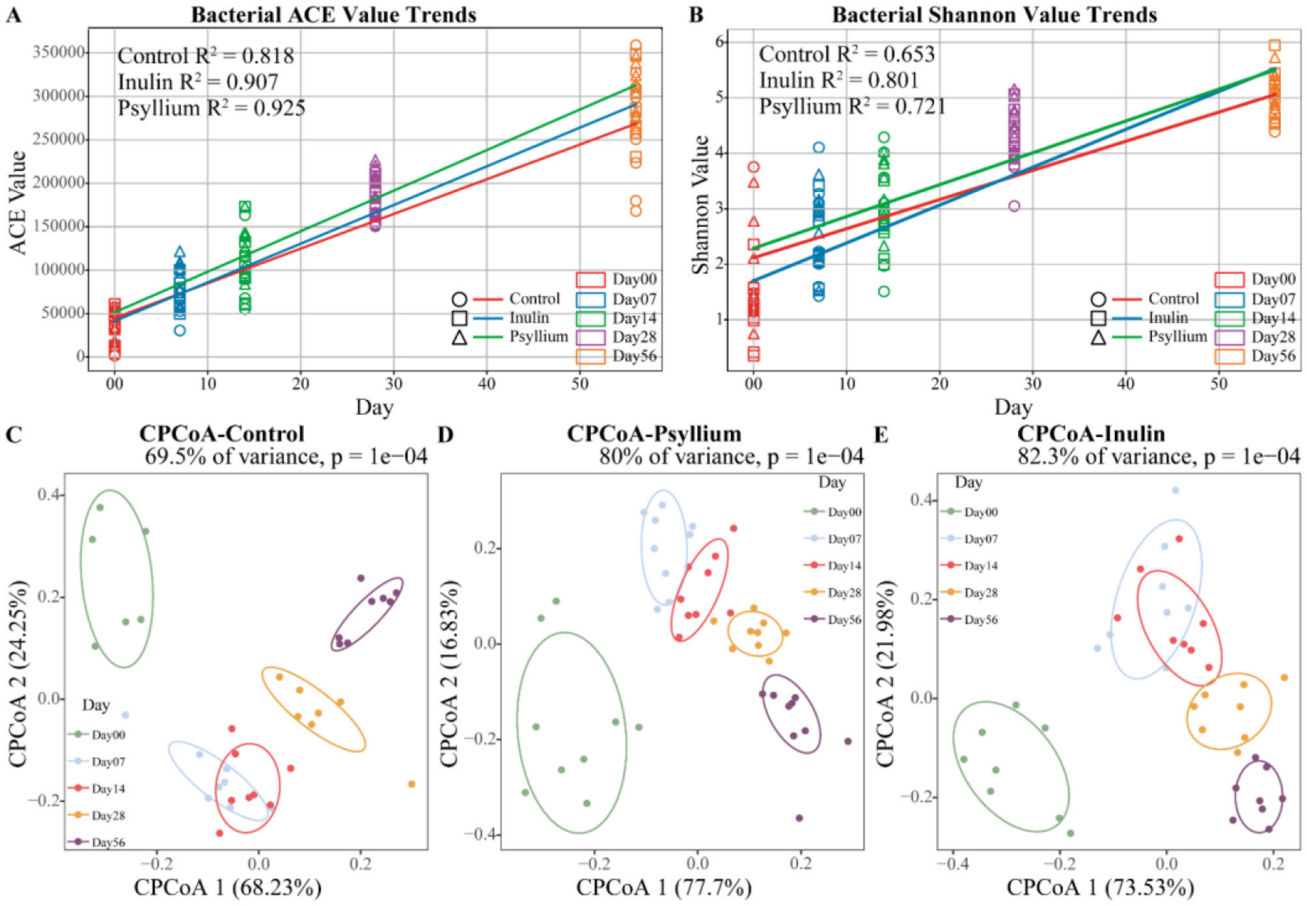
Dynamic analysis of fecal microbial diversity in calves across different ages. **(A, B)** Trends in the Shannon diversity index or ACE diversity index of fecal micro-organisms in calves from 0 to 56 days of age treated with dietary fiber. **(C–E)** CPCoA analysis of fecal microbial β-diversity in calves 0–56 days old in CON, PHP, and INU groups.

Dietary fiber supplementation significantly affected fecal microbiota diversity, with temporal variations in Shannon and ACE indices and *R*^2^ values exceeding 0.5 in linear regression analysis. At day 0, the structure of the fecal microbiota was relatively simple, with low α-diversity. However, the α-diversity of the fecal microbiota increased progressively with the calf's age. The fitted curves indicated that the ACE and Shannon indices in the INU and PHP groups were higher than those in the CON (control)group (Figures 1A, B). CPCoA revealed significant differences in the β-diversity of the fecal microbiota among the three groups over time (*P* < 0.01, Figures 1C–E). These findings suggest that the diversity and richness of the intestinal microbiota in pre-weaned calves changes significantly with increasing age.

### 3.2 Inulin and psyllium husk reshape fecal microbial structure in pre-weaning calves

**Figure 2:**
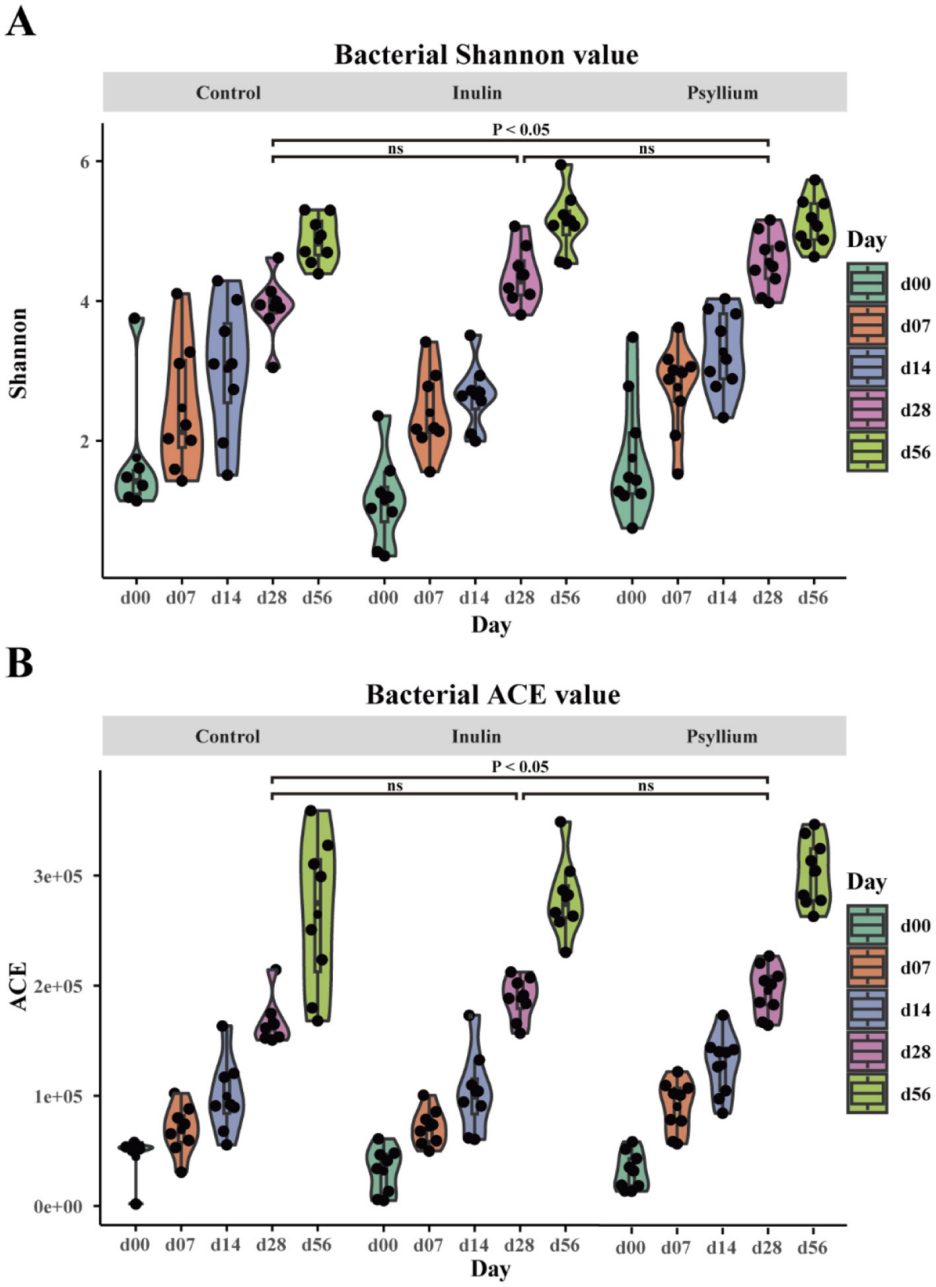
The impact of supplementation with psyllium husk powder and inulin on the α-diversity of the intestinal microbial community in neonatal calves. **(A)** Bacterial Shannon diversity index. **(B)** Bacterial ACE diversity index. **(A, B)** ANOVA (Analysis of Variance) with *post-hoc* Tukey HSD (Honestly Significant Difference) test, ns indicates no statistical difference, *P* < 0.05 indicates a significant statistical difference.

**Figure 3:**
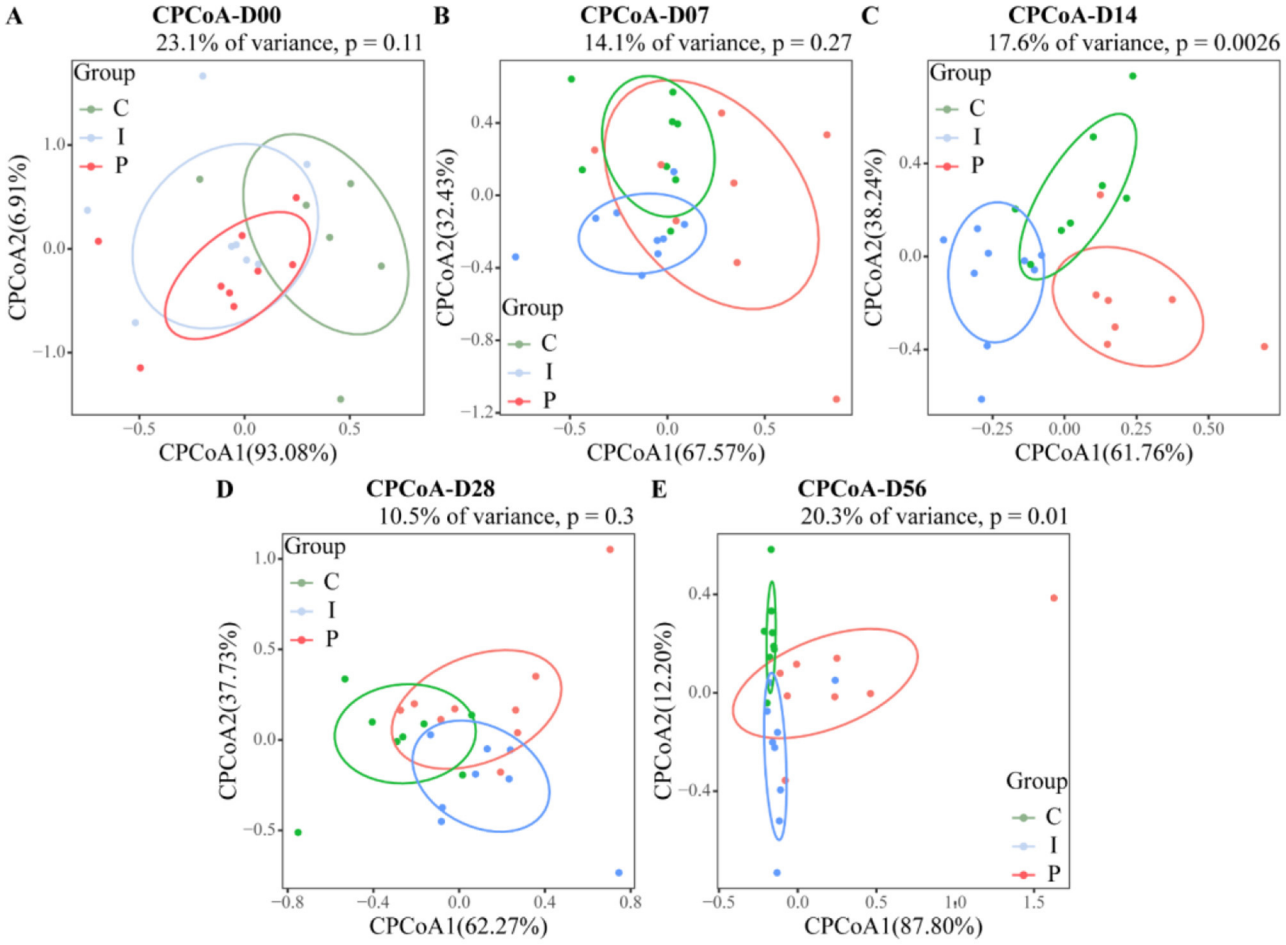
Assessment of the impact of psyllium husk powder and inulin supplementation on the β-diversity of the fecal microbiota in calves. **(A–E)** CPCoA analysis of fecal microbial β-diversity of CON, PHP, and INU calves at 0, 7, 14, 28, and 56 days. Group differences were calculated using the Chao distance, where *P* < 0.05 indicates a significant difference, and *P* < 0.01 indicates a highly significant difference.

Analysis of the Shannon and ACE indices revealed that at day 28, calves supplemented with psyllium husk powder had significantly higher Shannon and ACE indices, as compared to the control group (*P* < 0.05). In contrast, calves supplemented with inulin displayed no statistically significant differences from the control group, although the alpha diversity indices exhibited an increasing trend (Figure 2). CPCoA showed that intergroup differences became more pronounced at day 7, with an evident difference between the PHP and INU groups, indicating low similarity in the composition of the fecal microbiota between the two groups (Figures 3A, B). At day 14, significant differences in the composition of the fecal microbiota were found among the PHP, INU, and control groups (*P* < 0.01, Figure 3C). By day 28, the differences in the composition of the fecal microbiota among the three groups diminished (Figure 3D). However, at day 56, significant differences in the fecal microbiota composition were observed among calves supplemented with various dietary fibers (*P* < 0.05, Figure 3E). These findings suggested that dietary fiber supplementation in pre-weaned calves may alter the composition of the intestinal microbiota between days 14 and 56.

### 3.3 Dietary fiber modifies the relative abundance of calf fecal microbiota

**Figure 4:**
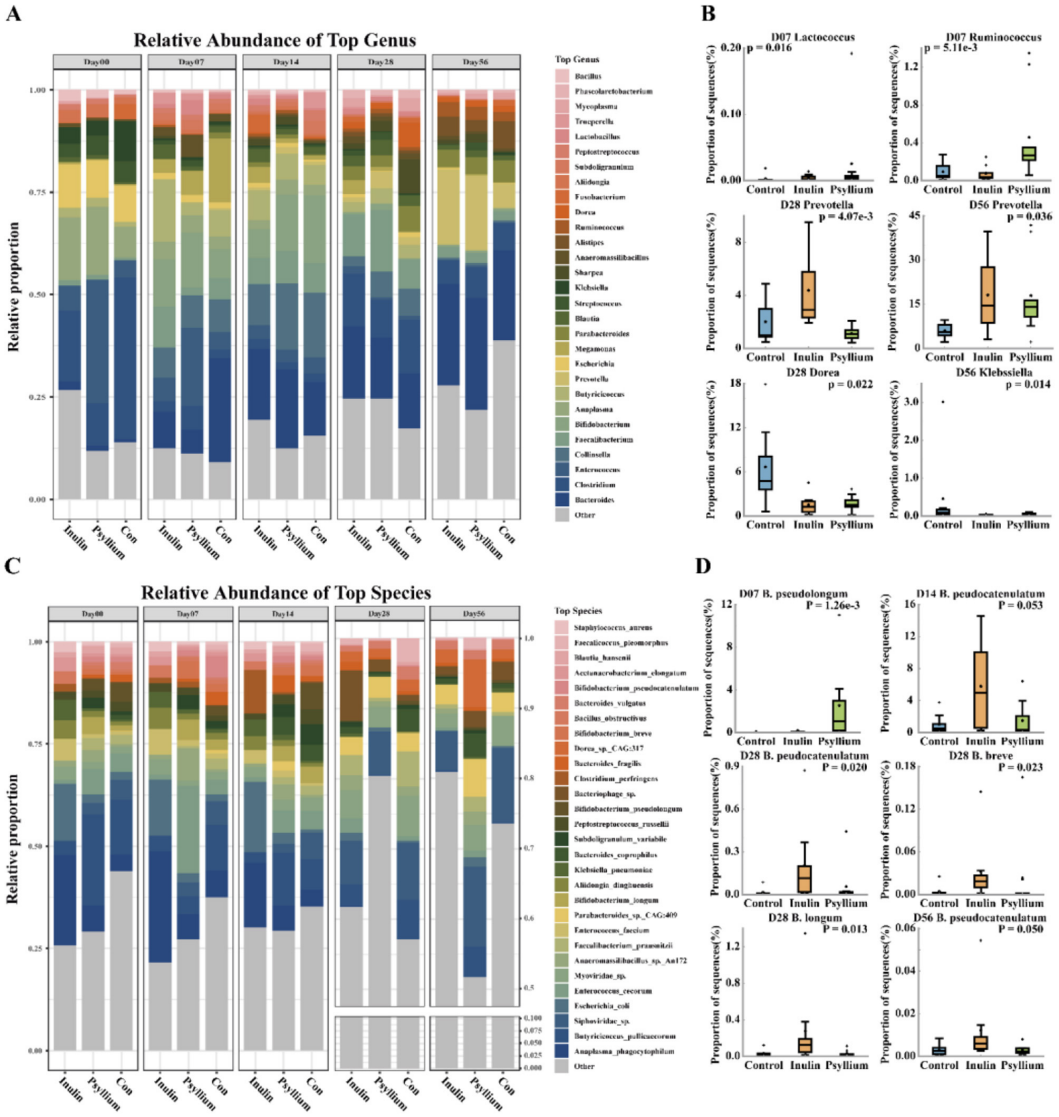
Analysis of the fecal microbiota at the genus and species levels in neonatal calves. **(A)** Stacked bar chart showing the relative abundance of species at the genus level. **(B)** Comparison of specific genera, including Lactococcus and Ruminococcus on day 7, *Prevotella* on day 28 and day 56, Dorea on day 28, and Klebsiella on day 56. **(C)** Stacked bar chart illustrating the relative abundance of species at the species level. **(D)** Differential abundance of Bifidobacterium species in calves of different ages and treatments (CON group, INU group, and Psyllium husk group). **(C, D)** Intergroup differences were calculated using the Kruskal–Wallis test, where *P* < 0.05 indicates a significant difference and *P* < 0.01 indicates a highly significant difference.

In this study, the 29 most abundant species were listed at the genus and species levels, and the remaining species with lower relative abundances were classified under the “Others” category. Relative abundance analysis at the genus level showed that the relative abundance of *Bacteroides* in the INU and PHP groups was lower than that in the control group at day 7. As the calf aged, the relative abundance of *Bacteroides* in the INU and PHP groups gradually increased, and after 28 days, the relative abundance of *Bacteroides* exhibited a trend of surpassing that of the control group (Figure 4A, Supplementary Figure S1). The relative abundance of *Enterococcus* tended to decrease during calf development, and the rate of decrease in the INU and PHP groups was slower than that in the control group. In addition, the relative abundance of *Enterococcus* in the PHP group was significantly increased as compared to that in the control and INU groups at day 28 (Figure 4A, Supplementary Figure S2A). The relative abundance of *Bifidobacterium* was significantly greater at days 7 and 14 after birth than at days 28 and 56. The relative abundance of *Bifidobacterium* in the INU group was substantially greater than that in the control group at days 7, 14, and 28. The relative abundance of *Bifidobacterium* in the PHP group was slightly lower than that in the INU group at days 7 and 14 (Figure 4A, Supplementary Figure S1).

Further analysis at the species level showed that at day 7 post-birth, the relative abundance of *Bifidobacterium pseudopodium* in the PHP group was significantly increased, as compared to that in the INU and control groups (*P* < 0.01). At day 14 after birth, the relative abundance of *Bifidobacterium pseudocatenulatum* in the INU group was greater than that in the PHP and control groups (*P* = 0.053). At day 28 post-birth, the relative abundances of *Bifidobacterium longum, Bifidobacterium pseudocatenulatum*, and *Bifidobacterium breve* in the INU group were substantially increased, as compared to those in the PHP and controls groups (*P* < 0.05, Figures 4C–D). The relative abundance of *Prevotella* in the intestine of neonatal calves was initially low, but gradually increased as the calves grew and developed (Figure 4A, Supplementary Figure S1). Moreover, on day 28, the relative abundance of *Prevotella* in the INU group was significantly greater than that in the control and PHP groups (*P* < 0.01). On day 56 after birth, the relative abundance of *Prevotella* in both the INU and PHP groups was significantly greater than that in the control group (*P* < 0.01, Figure 4B). Additionally, the relative abundance of *Lactococcus* in the control group was significantly lower than that in the INU and PHP groups (*P* < 0.05) on day 7. On day 288, the relative abundance of *Dorea* in the control group was significantly greater than that in the INU and PHP groups (*P* < 0.05), and on day 56, the relative abundance of *Klebsiella* in the control group was significantly increased, as compared to that in the INU and PHP groups (*P* < 0.05). Furthermore, on day 7, the relative abundance of *Ruminococcus* in the PHP group was significantly greater than that in the control and INU groups (*P* < 0.05, Figure 4B).

Further analysis at the species level revealed that on day 28, the relative abundance of *Blautia hansenii* in the INU group was significantly greater than that in the control and PHP groups (*P* < 0.05). In addition, the relative abundance of *Enterococcus faecium* in the PHP group was significantly increased as compared to that in the control and INU groups (*P* < 0.05). In contrast, the relative abundance of *Dorea* sp. CAG:317 in the control group was significantly greater than that in the INU and PHP groups (*P* < 0.05, Supplementary Figure S1B). On day 56, the relative abundance of *Siphoviridae* sp. in the control group was significantly increased as compared to that in the INU and PHP groups (*P* < 0.05), and the relative abundance of *Sharpea azabuensis* in the INU group was considerably greater than that in the control group (*P* < 0.05). In comparison, the relative abundance of *Sharpea azabuensis* in the PHP group was greater than that in the control group, but lower than that in the INU group. Additionally, the relative abundance of *Clostridium perfringens* in the control and INU groups was substantially lower than that in the PHP group (*P* < 0.05) (Supplementary Figure S1C).

**Figure 5:**
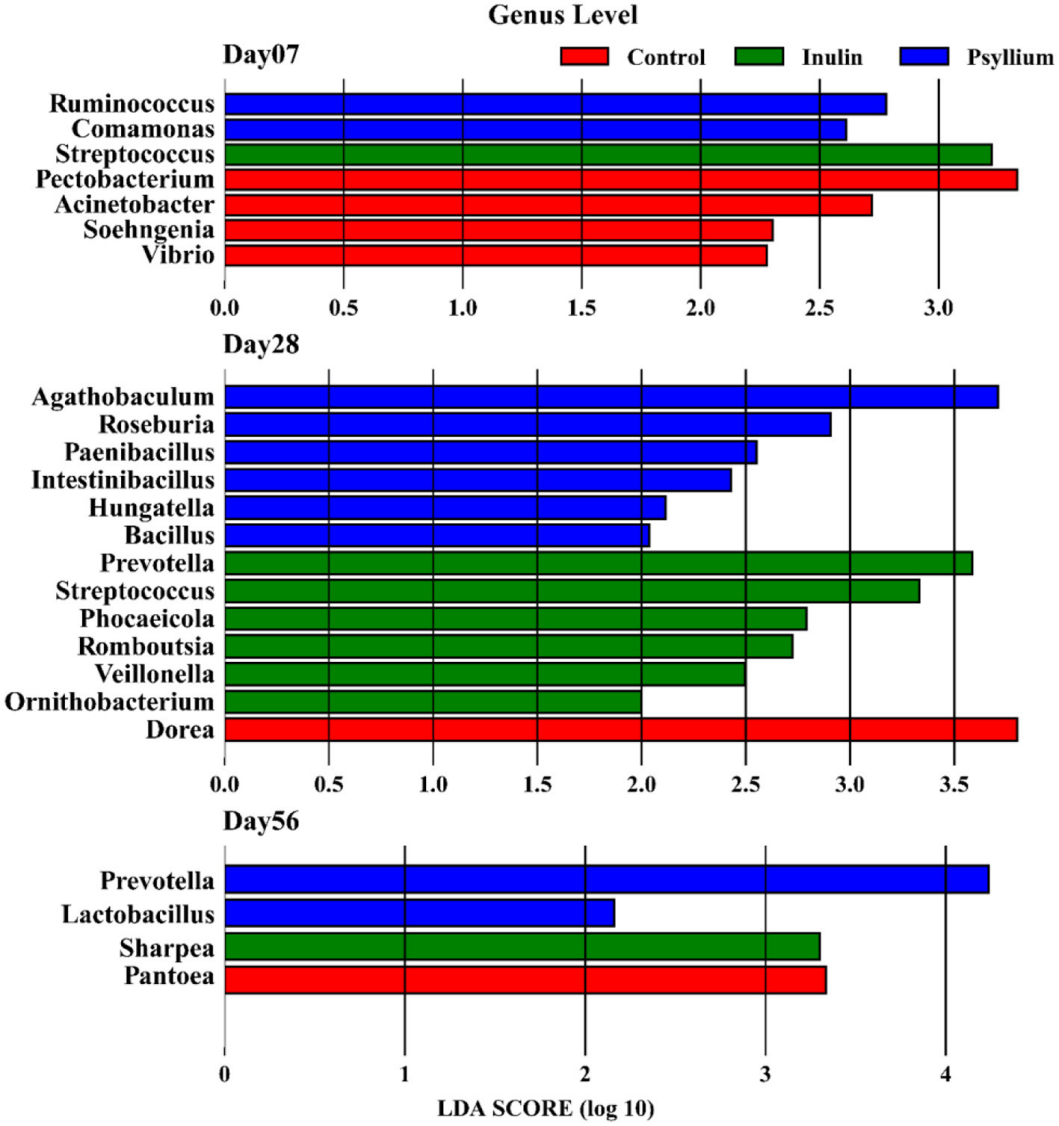
LEfSe analysis showing the differences in the microbiota composition at the genus level among the three groups (*P* < 0.05 and LDA > 2).

LEfSe (linear discriminant analysis effect size) analysis of the gut microbiota from calves supplemented with psyllium husk powder and inulin demonstrated significant differences at 7 days after birth. The abundance of *Ruminococcus* in the intestine of calves supplemented with psyllium husk powder significantly differed from that in the control group, while the abundance of *Streptococcus* in the INU group was significantly different (all *P* < 0.05, LDA > 2.0). In addition, there were significant differences in the abundances of the genera *Pectobacterium, Acinetobacter*, and *Vibrio* in the intestines of calves in the control group, as compared with those in the PHP and INU groups (all *P* < 0.05, LDA > 2.0). At day 28 after birth, the primary differential bacterial communities in the intestines of calves supplemented with psyllium husk powder were *Agathobaculum, Roseburia, Paenibacillus*, and *Intestinibacillus* (all *P* < 0.05, LDA > 2.0). In contrast, the major differential bacterial communities in the intestines of calves supplemented with inulin were *Prevotella, Streptococcus*, and *Phocaeicola* (all *P* < 0.05, LDA > 2.0). At day 56 post-birth, *Prevotella* was revealed as a significantly differential bacterial genus in the intestines of calves supplemented with psyllium husk powder (all *P* < 0.05, LDA > 4.0), and *Lactobacillus* also showed significant differences (all *P* < 0.05, LDA > 2.0). In the intestines of calves supplemented with inulin, *Sharpea* was found to be the primary bacterial group (all *P* < 0.05, LDA > 2.0, Figure 5).

LEfSe analysis at the species level revealed that *Bifidobacterium pseudolongum* was significantly differentially abundant in the intestinal tract of 7-day-old calves supplemented with inulin (all *P* < 0.05, LDA > 2.0). As the calves developed to 28 days of age, *Clostridium* sp-WCA-389-WT-23D1, *Agathobaculum desmolans*, and *Megamonas funiformis* were identified as the major differential microbiota in the PHP group, while *Prevotella copri* and *Prevotella sterecorea* in the *Prevotella* genus, *Bacteroides coprocola, Bacteroidaceae bacterium*, and *Bacteroides stercori* in the *Bacteroides* genus, and *Bifidobacterium longum* and *Bifidobacterium pseudocatenulatum* in the *Bifidobacterium* genus were identified as the differential microbiota in the INU group. Additionally, *Dorea* sp. CAG:317 and *Dorea phocaeensiss* were identified as a differential species in the control group (all *P* < 0.05, LDA > 2.0). *Prevotella* sp. P2-180 and *Prevotella stereorea* in the *Prevotella* genus were identified as differential species in the PHP group up until the calves reached 56 days of age. The primary differential species in the INU group was *Sharpea azabuensis* (all *P* < 0.05, LDA > 2.0, Supplementary Figure S2).

**Figure 6:**
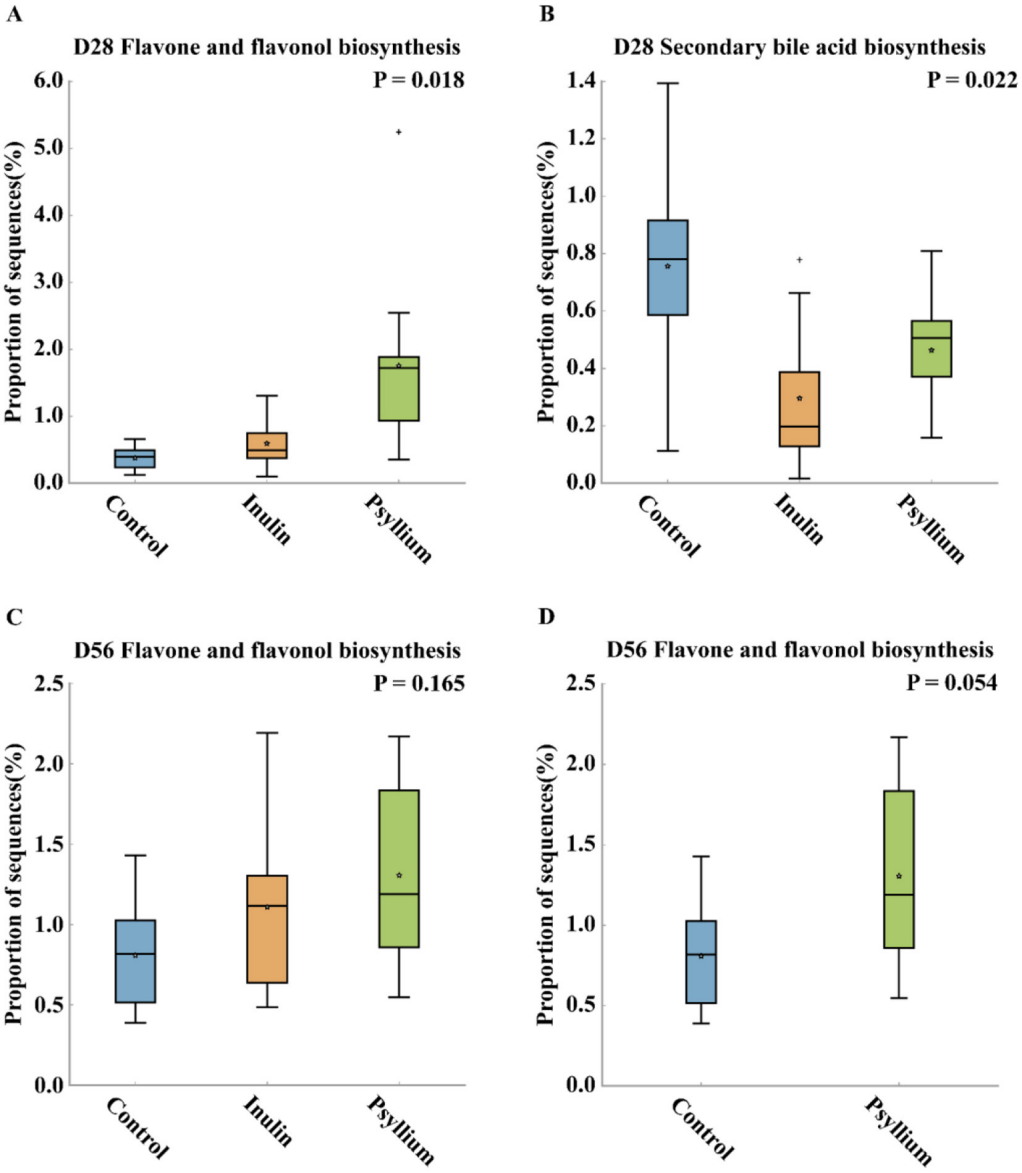
Illustrates the differential functions of the intestinal microbiota in the CON group, INU group, and Psyllium husk group. **(A)** Abundance of flavone and flavonol biosynthesis on day 28. **(B)** Abundance of secondary bile acid biosynthesis on day 28. **(C)** Abundance of flavone and flavonol biosynthesis on day 56. **(D)** Abundance of flavone and flavonol biosynthesis in the CON group and Psyllium husk group on day 56. *Data analysis was conducted using ANOVA.

At day 28 post-birth, the abundance of genes involved in the flavone and flavonol biosynthesis pathways in the PHP group was significantly increased, as compared to that in the control group (*P* < 0.05). However, at day 56 after birth, the flavone and flavonol biosynthesis pathways demonstrated an increase compared to those in the control group, but the difference was not statistically significant (Figures 6A, C–D). In the INU group, there was a slight increase in the flavone and flavonol biosynthesis pathways, as compared to that in the control group, but it was not statistically significant. However, the secondary bile acid biosynthesis pathway in the gut microbiota of calves in the INU group was significantly decreased, as compared to the control group (*P* < 0.05, Figure 6B).

**Figure 7:**
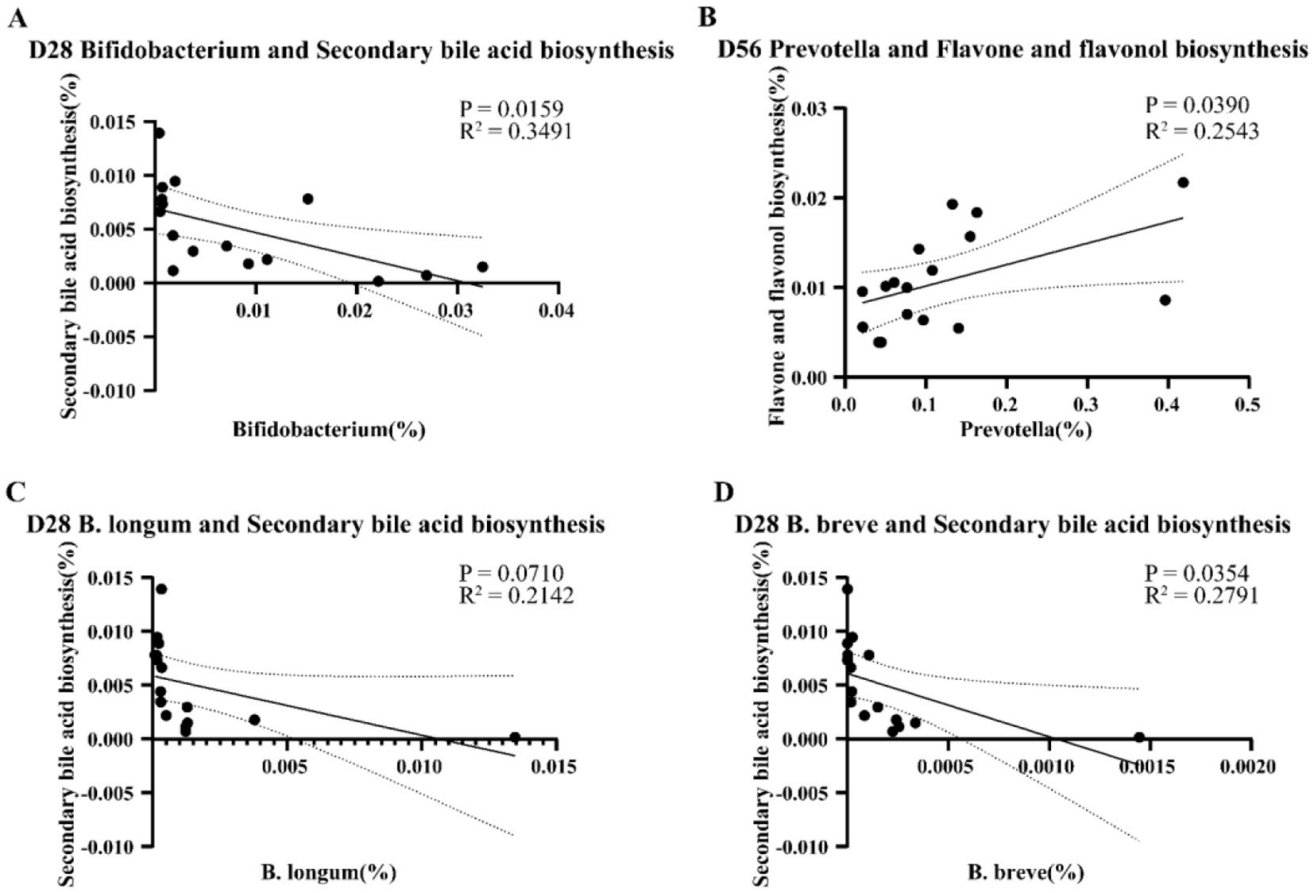
Presents the simple linear analysis of differential genera and differential functions. **(A)** The correlation between the Bifidobacterium genus and the secondary bile acid metabolism pathway on day 28. **(B)** The correlation between the *Prevotella* genus and flavone and flavonol biosynthesis on day 56. **(C)** The correlation between *B. longum* and secondary bile acid biosynthesis on day 28. **(D)** The correlation between *B. breve* and secondary bile acid biosynthesis on day 28.

We then conducted a simple linear analysis on the differential species and functions and found that on day 28, there was a significant negative correlation between *Bifidobacterium* spp. in the INU group and secondary bile acid biosynthesis pathway (*P* = 0.0159, *R*^2^ = 0.3491, Figure 7A). Further analysis at the species level revealed that *B. longum* was not significantly correlated with the secondary bile acid biosynthesis (*P* = 0.0710, *R*^2^ = 0.2142), but *B. breve* was significantly negatively correlated with secondary bile acid biosynthesis (*P* = 0.0354, *R*^2^ = 0.2791, Figures 7C, D). On day 56, there was a significant positive correlation between the abundance of *Prevotella* in the PHP group and the flavone and flavonol biosynthesis pathway (*P* = 0.0390, *R*^2^ = 0.2543, Figure 7B).

### 3.4 Comparative analysis of gut microbiota and CAZymes functions in PHP and INU

CAZymes families have been identified as vigorous predictors of animal diet. In order to investigate the potential influence of various dietary fibers on the gut microbiota of pre-weaned calves, we conducted STAMP (statistical analysis of taxonomic and functional profiles) differential analysis of CAZymes functions across the three groups of calves. We found that as the calves developed, the functions of the gut microbiota in the INU and PHP groups gradually increased, as compared to those of the control group. On day 14 of calf development, the INU group exhibited significant differences in the CAZymes function of their microbiota, as compared to the control group, with 6 CHs (Carbohydrate Hydrolases), 1 CBM (Carbohydrate-Binding Module), and 1 GT (Glycosyltransferases). By day 28 of calf development, the INU group showed further significant differences, with 10 CHs, 4 CBMs, 1 GT, and 1 PL (Polysaccharide Lyases). After the calves reached 56 days of age, the INU group exhibited substantial significant differences, with 20 CHs, 1 GT, and 2 CEs (Carbohydrate Esterases) (Supplementary Figure S3). At 14 days of age, calves in the PHP group exhibited significant differences from those in the control group, with 3 CHs, 3 GTs, and 2 CBMs. These significant differences increased by 28 days of age, with 5 CHs, 2 GTs, 3 CBMs, and 1 CE. At 56 days of age, the function of the gut microbiota from calves in the PHP group significantly differed from that in the control group, with 16 CHs, 3 GTs, and 2 PLs (Supplementary Figure S4).

**Figure 8:**
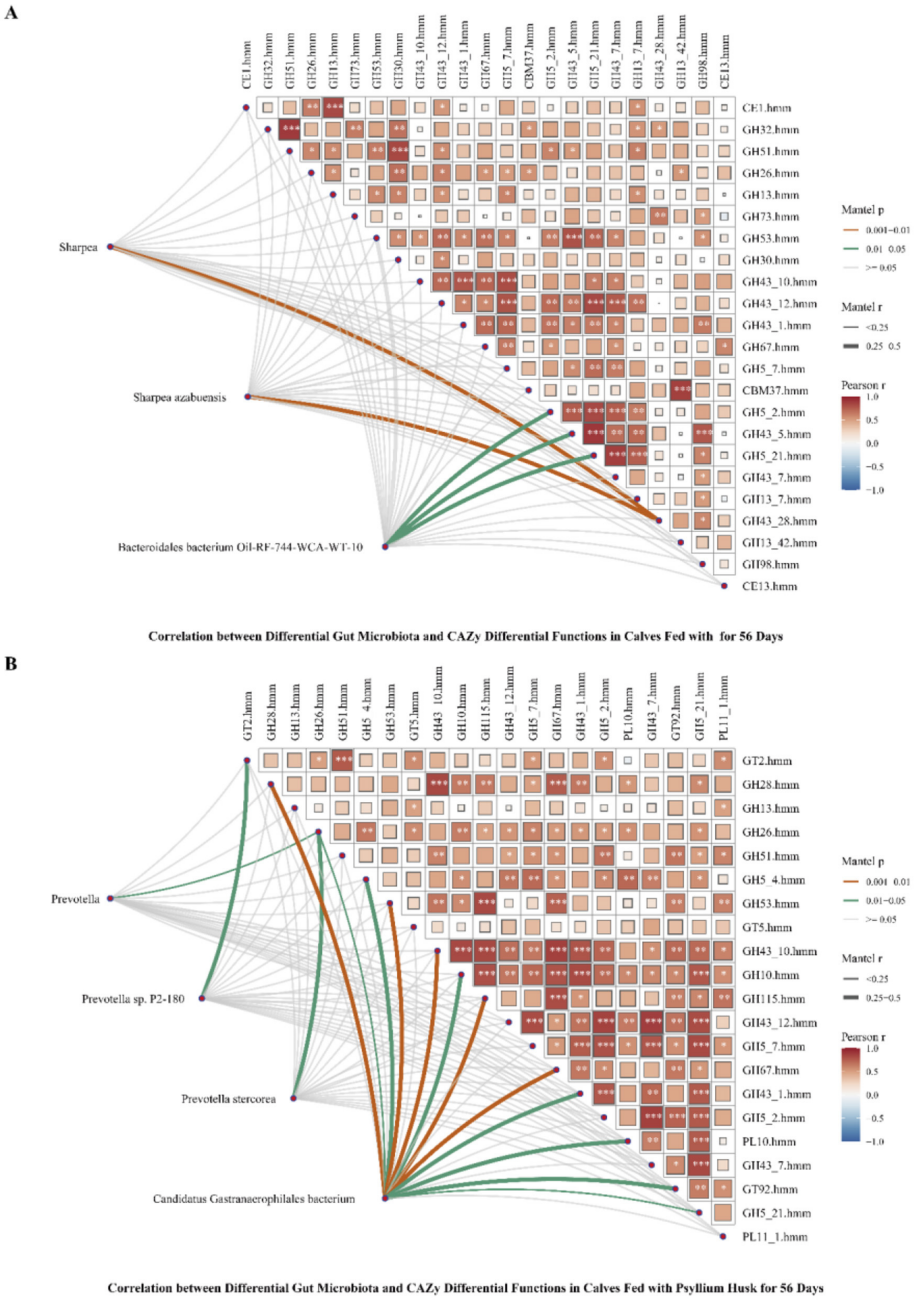
Association analysis of differential gut microbiota and CAZy differential functions in 56-day-old calves fed different dietary fibers. **(A)** Correlation between differential gut microbiota and CAZy differential functions in calves fed inulin for 56 days. **(B)** Correlation between Differential Gut Microbiota and CAZy Differential Functions in Calves Fed Psyllium Husk for 56 Days. **(A, B)** Correlation calculation using the Pearson algorithm, *P < 0.05, **P < 0.01, ***P < 0.001.

To investigate the potential associations between the intestinal microbiota of pre-weaned calves and differential functions of CAZymes in calves, we conducted a correlation analysis between the intestinal microbiota and differential functions of CAZymes in calves at D56. We found that regardless of whether the calves were fed inulin or psyllium husk powder, the functions of CAZymes in the gut microbiota of the calves were mostly positively correlated with bacterial genera and species (Figures 8A, B). There was a significant correlation between the intestinal microbiota of the *Sharpea* genus *Sharpea azabuensis* and GH43_28.hmm in calves fed inulin (*P* < 0.01, *R* > 0.25). The abundances of the *Bacteroidales* bacterium Oil-RF-744-WCA-WT-10 were significantly correlated with the abundances of GH5_2.hmm, GH43_5.hmm, and GH5_21.hmm (*P* < 0.05, *R* > 0.25). There was a substantial correlation between the abundance of the *Prevotella* genus *Prevotella* sp. P2-180 and that of GT2.hmm in the gut microbiota of calves fed psyllium husk powder (*P* < 0.05, *R* > 0.25). *Prevotella stereorea* exhibited a significant correlation with GH26.hmm (*P* < 0.05, *R* > 0.25). *Candidatus Gastranaerophilales* showed a highly significant correlation with 5 GHs (*P* < 0.01, *R* > 0.25), a significant correlation with 4 GHs (*P* < 0.05), and a significant correlation with PL10.hmm and GT92.hmm (*P* < 0.05, *R* > 0.25).

### 3.5 Impact of dietary fiber on gut microbial symbiosis in 56-day-old calves

**Figure 9:**
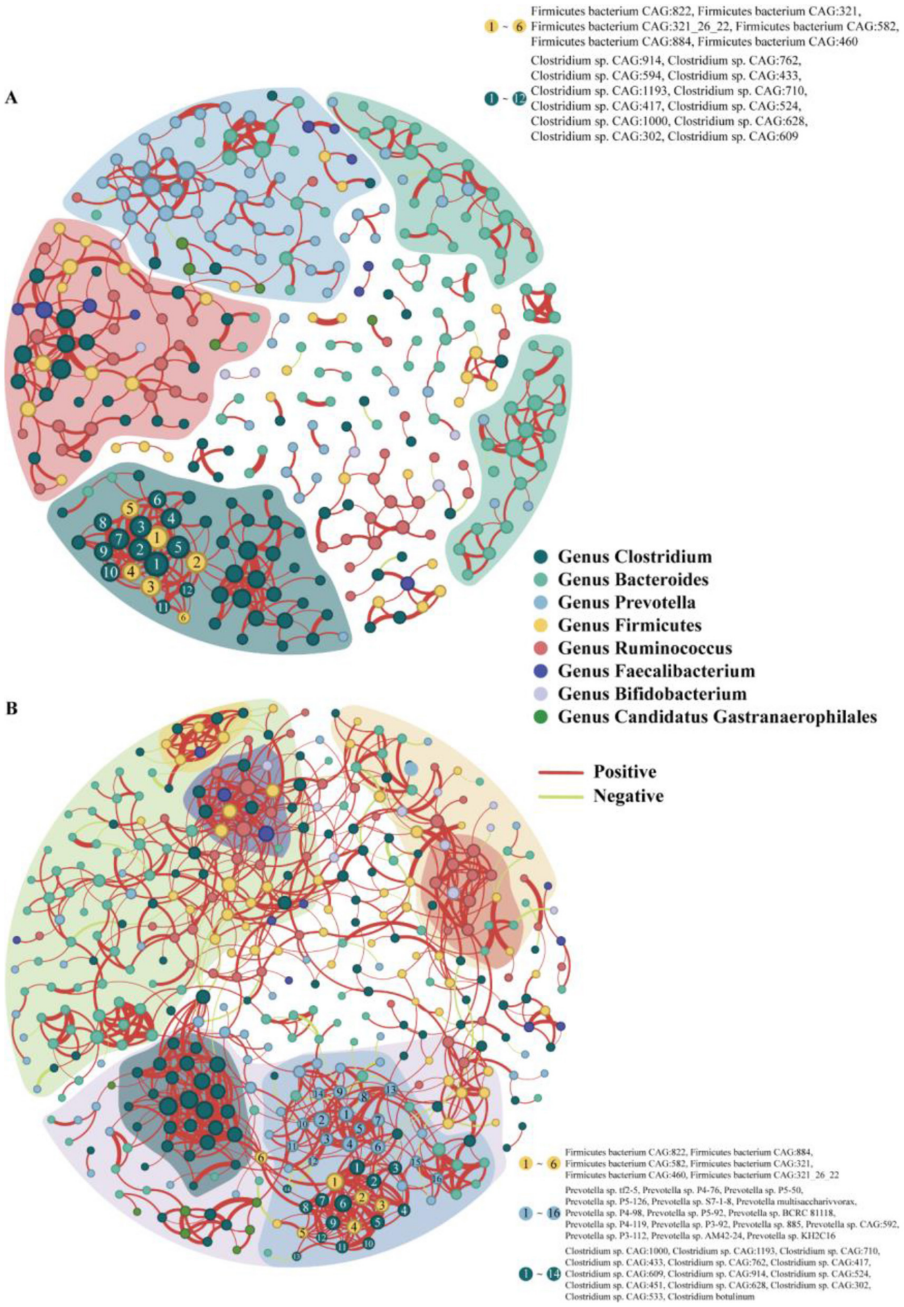
Co-occurrence network of intestinal microbiota at the species level in **(A)** 56-day-old calves supplemented with inulin and **(B)** 56-day-old calves supplemented with Psyllium husk. Each node represents a species, and the edges represent a significant co-occurrence relationship. The top 400 correlating nodes are included in the graph (those with correlations <400 are the nodes with the highest correlation).

We analyzed the symbiotic relationships within the intestinal microbiota of 56-day-old calves and found that calves supplemented with inulin displayed weak intergenus associations, with only the *Prevotella, Clostridium*, and *Bacteroides* genera showing relatively strong intragenus correlations (Figure 9A). Conversely, calves supplemented with psyllium husk powder displayed strong intergenus associations within their intestinal microbiota. We also observed that *Prevotella, Clostridium, Firmicutes, Bacteroides*, and *Candidatus Gastranaerophilales* formed an intergenus symbiotic cluster, while *Bacteroides, Faecalibacterium*, and *Ruminococcus* formed another intergenus symbiotic cluster. Additionally, the abundances of *Prevotella, Clostridium*, and *Firmicutes* exhibited relatively high intra-genus correlations (Figure 9B).

## 4 Discussion

Diarrhea is a major health challenge in neonatal calves, causing high morbidity, mortality, and economic losses, while dietary fiber supports gastrointestinal health by alleviating diarrhea, constipation, and inflammation (Foster and Smith, 2009; Shoaib et al., 2016; Urie et al., 2018; Belorio and Gómez, 2021). Our findings suggest that supplementing neonatal calves with psyllium husk powder or inulin accelerates the establishment of a beneficial intestinal microbiota. Twenty-eight days after birth, calves in the psyllium husk group exhibited significantly greater microbial diversity and richness as compared to the control group. Furthermore, we found that the intestinal microbiota transitioned between days 14 and 28, highlighting a critical period of microbial maturation and underscoring the potential of dietary fiber intervention in supporting intestinal health during early development.

To explore the effects of psyllium husk powder and inulin on the gut microbiota of calves, we employed metagenomic analysis to assess variations in distinct microbial communities within the intestines of calves across various developmental stages. During the early stages of preweaning calf development, alterations in the gut microbiota induced by feeding psyllium husk powder or inulin were not prominent. However, *Ruminococcus* and *Streptococcus* were identified as differential microbial communities in the intestines of calves supplemented with dietary fiber. It has been previously reported that *Ruminococcus* is a critical symbiotic bacterium and butyrate-producing bacterium capable of fermenting plant fiber to produce short-chain fatty acids (La Reau and Suen, 2018). Moreover, *Ruminococcus* is a vital producer of butyrate, a beneficial short-chain fatty acid. During the early stages of calf development, supplementation with psyllium husk powder resulted in an increased abundance of *Ruminococcus*, suggesting that *Ruminococcus* could ferment psyllium husk powder to produce short-chain fatty acids. At day 28, as the calf intestinal microbiota diversity increased, beneficial bacteria, such as *Agathobaculum, Roseburia*, and *Prevotella*, became the dominant differential genera in the intestines of calves supplemented with psyllium husk powder and inulin. By day 56, the gut microbiota had stabilized. In calves supplemented with psyllium husk powder, *Prevotella* and *Lactobacillus* were the dominant genera. Additionally, *Ruminococcus, Agathobaculum*, and *Roseburia*—known butyrate producers—played a crucial role in providing energy for intestinal epithelial cells, supporting calf development (Rivière et al., 2016). Additionally, butyrate displays immunomodulatory and anti-inflammatory properties, aiding in preserving intestinal barrier function, which is crucial for enhancing resistance to diarrhea in neonatal calves. Furthermore, numerous studies have shown that feeding dietary fiber, such as inulin, increases the abundance of the *Bifidobacterium* genus. Concurrently, *Bifidobacterium* interacts with butyrate-producing bacteria, thereby promoting butyrate production. In this study, calves fed psyllium husk powder, exhibited a substantial increase in the abundance of *Bifidobacterium pseudolongum* at day 7, while calves fed inulin showed significant differences in the abundances of *Bifidobacterium breve, Bifidobacterium longum*, and *Bifidobacterium pseudocatenulatum* at days 14, 28, and 56, respectively. This finding indicates that both psyllium husk powder and inulin may have bifidogenic effects. In the intestines of calves fed psyllium husk powder, a significant increase in the abundance of the *Bifidobacterium* genus was not observed, but differences in the butyrate-producing bacteria were evident. We theorize that psyllium husk powder stimulates an increase in the abundance of butyrate-producing bacteria through other pathways, thereby promoting butyrate production.

Differential analysis of KEGG pathways identified a significant increase in flavone and flavonol biosynthesis pathways in calves supplemented with psyllium husk powder. As the calves reached 56 days of age, the activity of the flavone and flavonol biosynthesis pathway was elevated compared to the control group, but the difference was not statistically significant. Compared with the control group, the secondary bile acid biosynthesis pathway in the calves, supplemented with inulin, was significantly decreased. Furthermore, simple linear regression analysis indicated that the abundance of *Bifidobacterium* at day 28 was significantly correlated with the secondary bile acid biosynthesis pathway in calves supplemented with inulin. At the species level, *B. breve* was significantly correlated with the secondary bile acid biosynthesis pathway, while *B. longum* exhibited some correlation with the secondary bile acid biosynthesis pathway, although the correlation was not statistically significant. These results indicate that the increased abundance of *Bifidobacterium* partially reduces the activity of the secondary bile acid biosynthesis pathway. Previous studies have demonstrated that the metabolic products synthesized by the secondary bile acid biosynthesis pathway are related with the development of fatty liver, obesity, and colorectal cancer in humans and animals (Jia et al., 2021; Liu et al., 2022). Current research indicates that deoxycholic acid, a metabolic product involved in the secondary bile acid biosynthesis pathway, adversely alters the barrier function of the intestinal epithelium (Stenman et al., 2013; Liu et al., 2018; Zhuang et al., 2024). Our study suggests that supplementing pre-weaned calves with inulin enriches *Bifidobacterium* during the early development of the intestinal barrier, which inhibits the secondary bile acid biosynthesis pathway to protect intestinal health and promotes intestinal recovery, thereby reducing the incidence of calf diarrhea.

When the calves reached 56 days of age, the structure of their gut microbiota closely resembled that of adult cattle. Our investigation revealed substantial differences at day 56 in the abundance of *Prevotella* between calves supplemented with psyllium husk powder and those in the control group. Through species-level LEfSe analysis, we identified *Prevotella* sp. P2-180 and *Prevotella stereorea* as the primary differential strains. Further simple linear regression analysis identified a significant positive correlation between the abundance of *Prevotella*, as well as the flavone and flavonol biosynthesis pathways. This study confirms that both flavone and flavonol biosynthesis, both of which produce flavonoid compounds that play various roles in the gastrointestinal tract, are essential metabolic pathways in the gut microbiota. These pathways perform essential functions, including maintaining intestinal barrier integrity, modulating intestinal immune system, regulating intestinal hormone secretion, promoting beneficial microbial communities, inhibiting harmful endotoxin production, and positively regulating beneficial fatty acid production (Oteiza et al., 2018). Furthermore, prior research suggests that intestinal microbiotas abundant in *Prevotella* possess an enhanced capacity for the utilization of complex polysaccharides (Chen et al., 2017), which aligns with our findings, indicating a significant increase in *Prevotella* during the later stages of preweaning in calves supplemented with psyllium husk powder or inulin, as compared to the control group. *Prevotella* is an intestinal probiotic strongly correlated with the production of total short-chain fatty acids. A previous *in vitro* study suggested that supplementing a diet with flavonoids and dietary fibers that produced SCFAs (Short-chain fatty acids) significantly enriched *Prevotella* (Lucas et al., 2018). This preliminary study also suggested that flavonoids could promote SCFAs production via *Prevotella*. We speculated that supplementation with psyllium husk powder in calves at 28 days of age gradually activated the flavone and flavonol biosynthesis pathways in the intestinal microbial community, leading to the production of flavonoids. These flavonoids promote the colonization of *Prevotella* in the intestines of calves, as well as the production of SCFAs. Both *Prevotella* and SCFAs protected the health of post-weaning calf intestinal epithelial cells, maintained intestinal metabolic health, and preserved intestinal mucosal barrier integrity, thereby maintaining intestinal homeostasis (Xiong et al., 2019; Blaak et al., 2020; Silva et al., 2020).

The physiological functions of the bovine digestive system differ considerably from those of monogastric animals. Adult cows rely on the gastrointestinal microbiota to ferment cellulose and other substrates, producing volatile fatty acids to meet their energy requirements. The digestive system of pre-weaned calves is similar to that of monogastric animals, but as weaning approaches, the rumen of calves begins to progressively develop, transitioning toward that of adult cattle. Our study demonstrated significant differences at day 56 in the intestinal microbiota of calves fed inulin, particularly in the abundance of the *Sharpea* genus and *Bacteroidales* bacterium Oil-RF-744-WCA-WT-10. These differences were significantly correlated with enzymes from the GH43 and GH5 families. Previous research has suggested that the *Sharpea* genus is an important producer of lactic acid and can work synergistically with other associated genera to promote the production of butyrate (Kamke et al., 2016; Lin et al., 2019; Zhang et al., 2022). Additionally, this genus stabilizes the ecosystem of the intestinal microbiota, leading to increased production of butyrate for intestinal epithelial cell function, maintaining intestinal barrier integrity, and laying the foundation for the maturation of the calf's digestive system.

In calves supplemented with Psyllium husk, the *Prevotella* genus was the primary differential species at day 56. Pearson correlation analysis revealed significant correlations between the *Prevotella* genus and enzyme families, including GT2 and GH26. *Candidatus Gastranaerophilales* bacteria were found to be significantly correlated with the enzyme families GH5, GH10, GH26, GH28, GH43, GH53, GH67, GH115, GT92, and PL10. Previous studies have indicated that *Candidatus Gastranaerophilales* bacteria contribute to the production of indole, which can be converted into indole-3-propionic acid (IPA). IPA has been shown to play an anti-inflammatory role in the gastrointestinal tract and throughout the body, thus protecting the intestinal health of calves and reducing diarrhea (Zhao et al., 2019; Rosario et al., 2021).

According to the CAZy database (https://www.CAZy.org) classification, enzymes involved in carbohydrate metabolism are categorized as glycosyl hydrolases (GHs), polysaccharide lyases (PLs), carbohydrate esterases (CEs), and glycosyl transferases (GTs). These enzymes display substrate specificity in carbohydrate degradation (Helbert et al., 2019). Previous studies have demonstrated that the intake of different forms of carbohydrates can promote specific bacterial taxa and alter the CAZymes profile in the intestine (Cronin et al., 2021; Ye et al., 2022). In this study, the intestinal microbiota of calves, supplemented with fructooligosaccharides (FOSs) and psyllium husk powder before weaning, showed increased levels of multiple CAZymes families, as compared with those in the control group. Specifically, at day 56 the FOSs group showed significant increases in enzyme families GH5, GH13, GH26, GH30, GH32, GH43, GH51, GH53, GH67, GH73, GH98, and CE13, as compared to the control group, while the PHP group showed significant increases in families GT2, GT5, GT92, GH5, GH10, GH13, GH26, GH28, GH43, GH51, GH53, GH67, GH115, PL10, and PL11, as compared to the control group. We hypothesized that due to the different stimulating effects of FOS as a single-substrate dietary fiber and psyllium husk powder, as a complex dietary fiber on the CAZymes of the intestinal microbiota, these CAZymes could influence polysaccharide digestion, which supported the dietary structure transition in calves after weaning. Additionally, the promotion of different intestinal bacterial populations in calf intestines may be facilitated by various substrates.

**Figure 10:**
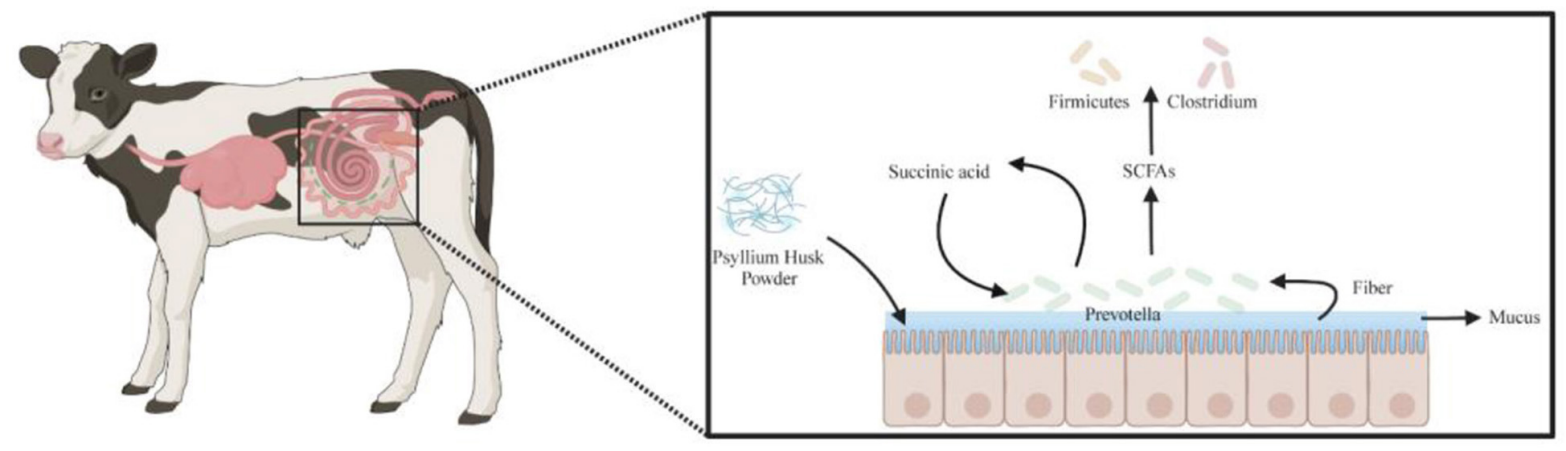
Schematic representation of symbiotic gut microbiota and metabolic pathways in calves (Created in https://BioRender.com).

Our study found that supplementation with different dietary fibers significantly influenced the fecal microbiota composition and functional metabolism in calves. In the PHP group, the dominant symbiotic bacterial community at the genus level comprised *Prevotella, Clostridium*, and *Firmicutes*, exhibiting a unique metabolic synergy. Studies have shown that certain *Clostridium* and *Firmicutes* strains (e.g., *Clostridium* sp. CAG:914, *Clostridium* sp. CAG:302, *Firmicutes bacterium* CAG:321, and *Firmicutes bacterium* CAG:884) are negatively correlated with short-chain fatty acids (SCFAs) levels (Zhao et al., 2022), suggesting that these strains may act as SCFAs consumers. In contrast, *Prevotella* strains (e.g., *Prevotella* sp. *TF2-5* and *Prevotella* sp. P5-50) decompose plant fibers to generate SCFAs (Accetto and Avguštin, 2019), while *Prevotella* sp. P4-76 enhances mucin-layer carbohydrate metabolism and promotes succinate production, which is subsequently converted into propionate (an SCFA) via the succinate-propionate pathway (Le Bastard et al., 2020b). A key role of psyllium husk powder lies in its ability to stimulate mucin production (Martellet et al., 2022), thereby providing abundant metabolic substrates for *Prevotella* strains, significantly enhancing their carbohydrate-degrading capacity and increasing SCFA and succinate production. For instance, *Prevotella* sp. *AM42-24* exhibits polysaccharide degradation and fermentation capabilities in the rumen (Shinkai et al., 2022), while *Prevotella multisaccharivorax*, a dominant species in the calf gastrointestinal tract, primarily ferments carbohydrates to produce acetate and succinate (Wang et al., 2023), further highlighting the central metabolic role of *Prevotella* in the PHP group. These metabolic products are subsequently utilized by specific *Firmicutes* and *Clostridium* strains, forming a mutualistic symbiotic relationship: *Prevotella* acts as an SCFA “producer”, while certain *Firmicutes* and *Clostridium* strains function as “consumers”, depending on these metabolic products for growth. This producer-consumer synergy contributes to the stabilization of the intestinal microenvironment (Figure 10). In contrast, the INU group exhibited a weaker symbiotic relationship among *Prevotella, Clostridium*, and *Firmicutes*, lacking the same level of metabolic synergy observed in the PHP group. Therefore, psyllium husk plays a crucial role in modulating gut microbiota structure and function by promoting mucin production, enhancing *Prevotella* metabolic activity, and supporting the utilization of SCFAs by downstream microbial communities. This mechanism not only reveals the intrinsic symbiotic interactions within the PHP group but also provides a scientific basis for dietary fiber interventions aimed at improving gut health, highlighting the unique potential of psyllium husk in maintaining intestinal microbial homeostasis.

## 5 Conclusion

In conclusion, this study investigated the effects of inulin and psyllium husk powder supplementation on the composition of the intestinal microbiota in pre-weaned calves, as well as the KEGG and CAZymes functions of the intestinal microbiota. This study found that both inulin and psyllium husk powder increased the abundance of the intestinal microbiota to a certain extent, while also promoting the colonization of beneficial bacteria, such as *Bifidobacterium, Prevotella*, and *Sharpea* at various stages before weaning. It is theorized that *Bifidobacterium* protects the intestinal epithelium and preserves intestinal health by inhibiting the activity of the secondary bile acid metabolism pathway, whereas *Prevotella* produces flavonoid metabolites to preserve intestinal health by promoting the flavonoid metabolism pathway. Furthermore, supplementation with various dietary fibers resulted in upregulation of CAZymes in the calf intestinal microbiota, indicating that dietary fiber supplementation during the calf gastrointestinal transition stage may affect the carbohydrate degradation capacity of the intestines, which warrants further investigation into its influence on subsequent production performance. This study demonstrated the potential of supplementing pre-weaned calves with dietary fiber to improve the structure of the calf intestinal microbiota, promote the colonization of beneficial microorganisms, and protect intestinal health, thus providing deeper insights into the regulatory effects of various dietary fibers on the calf intestinal microbiota.

## Data Availability

The raw data supporting the findings of this study have been deposited in the National Microbiology Data Center (NMDC) under the Project ID: NMDC10019387. These data are publicly accessible and provide comprehensive information for reproducing the results reported in this study. Researchers interested in further analyses or validations are encouraged to utilize this dataset.
