# Supplementary material for: Effects of dietary fiber on the composition, function, and symbiotic interactions of intestinal microbiota in pre-weaned calves
Source: Front Microbiol. 2025 Mar 25;16:1554484. doi: 10.3389/fmicb.2025.1554484 (PMC11975667; doi:10.3389/fmicb.2025.1554484)
Supplement: Supplementary file 1 [file Data_Sheet_1.docx]

Supplementary Material

1. **Supplementary Data**

**Table S1: NGS Quality Control**

| NGS Quality Control | | | | | | | |
| --- | --- | --- | --- | --- | --- | --- | --- |
| Sample ID | | Total Reads | Clean Reads | Host Reads | Clean Precent | | Host Precent |
| C15d00 | | 17144668 | 12413066 | 4731602 | 72.40% | | 27.60% |
| C15d07 | | 20167004 | 13059196 | 7107808 | 64.76% | | 35.24% |
| C15d28 | | 13952384 | 7601150 | 6351234 | 54.48% | | 45.52% |
| C15d56 | | 36658398 | 22081768 | 14576630 | 60.24% | | 39.76% |
| C23d00 | | 20918428 | 20859388 | 59040 | 99.72% | | 0.28% |
| C23d07 | | 23938284 | 13414340 | 10523944 | 56.04% | | 43.96% |
| C23d14 | | 22446606 | 13116266 | 9330340 | 58.43% | | 41.57% |
| C23d28 | | 21644718 | 15191330 | 6453388 | 70.18% | | 29.82% |
| C23d56 | | 27301958 | 16124054 | 11177904 | 59.06% | | 40.94% |
| C24d00 | | 17117576 | 12312572 | 4805004 | 71.93% | | 28.07% |
| C24d07 | | 26071404 | 8799382 | 17272022 | 33.75% | | 66.25% |
| C24d14 | | 21080436 | 8359658 | 12720778 | 39.66% | | 60.34% |
| C24d28 | | 17530634 | 11651598 | 5879036 | 66.46% | | 33.54% |
| C24d56 | | 23473020 | 13971054 | 9501966 | 59.52% | | 40.48% |
| C25d07 | | 26304178 | 15221838 | 11082340 | 57.87% | | 42.13% |
| C25d14 | | 18171620 | 12781638 | 5389982 | 70.34% | | 29.66% |
| C25d28 | | 22117014 | 12116006 | 10001008 | 54.78% | | 45.22% |
| C25d56 | | 16715190 | 10907832 | 5807358 | 65.26% | | 34.74% |
| C26d00 | | 28159552 | 2928004 | 25231548 | 10.40% | | 89.60% |
| C26d07 | | 12464810 | 7969312 | 4495498 | 63.93% | | 36.07% |
| C26d14 | | 33261364 | 20293846 | 12967518 | 61.01% | | 38.99% |
| C26d56 | | 27340442 | 11837614 | 15502828 | 43.30% | | 56.70% |
| C27d00 | | 38303508 | 17053070 | 21250438 | 44.52% | | 55.48% |
| C27d07 | | 24885500 | 12351794 | 12533706 | 49.63% | | 50.37% |
| C27d14 | | 22436202 | 14125186 | 8311016 | 62.96% | | 37.04% |
| C27d28 | | 20310552 | 12228600 | 8081952 | 60.21% | | 39.79% |
| C27d56 | | 26023140 | 12914378 | 13108762 | 49.63% | | 50.37% |
| C30d00 | | 21863076 | 13230116 | 8632960 | 60.51% | | 39.49% |
| C30d07 | | 24076592 | 11704288 | 12372304 | 48.61% | | 51.39% |
| C30d14 | | 21900868 | 10868644 | 11032224 | 49.63% | | 50.37% |
| C30d28 | | 20177498 | 13780566 | 6396932 | 68.30% | | 31.70% |
| C30d56 | | 26662800 | 16342122 | 10320678 | 61.29% | | 38.71% |
| C36d28 | | 29310504 | 17464282 | 11846222 | 59.58% | | 40.42% |
| C38d07 | | 17922226 | 17196722 | 725504 | 95.95% | | 4.05% |
| C38d14 | | 2733210 | 1645034 | 1088176 | 60.19% | | 39.81% |
| C38d28 | | 19556590 | 12142936 | 7413654 | 62.09% | | 37.91% |
| C38d56 | | 23828938 | 3863338 | 19965600 | 16.21% | | 83.79% |
| I01d00 | | 22891854 | 16026864 | 6864990 | 70.01% | | 29.99% |
| I01d07 | | 28588052 | 11596586 | 16991466 | 40.56% | | 59.44% |
| I01d14 | | 25901554 | 9518584 | 16382970 | 36.75% | | 63.25% |
| I01d28 | | 28291918 | 15147334 | 13144584 | 53.54% | | 46.46% |
| I01d56 | | 27266806 | 14635012 | 12631794 | 53.67% | | 46.33% |
| I11d00 | | 21814606 | 10710644 | 11103962 | 49.10% | | 50.90% |
| I11d07 | | 19906156 | 14047726 | 5858430 | 70.57% | | 29.43% |
| I11d14 | | 21390748 | 15817604 | 5573144 | 73.95% | | 26.05% |
| I11d28 | | 24258262 | 16501604 | 7756658 | 68.02% | | 31.98% |
| I11d56 | | 30798358 | 16495964 | 14302394 | 53.56% | | 46.44% |
| I31d00 | | 11512350 | 9469338 | 2043012 | 82.25% | | 17.75% |
| I31d07 | | 23218394 | 12230266 | 10988128 | 52.67% | | 47.33% |
| I31d14 | | 22558088 | 11508774 | 11049314 | 51.02% | | 48.98% |
| I31d28 | | 25556708 | 12755406 | 12801302 | 49.91% | | 50.09% |
| I31d56 | | 26984016 | 13306218 | 13677798 | 49.31% | | 50.69% |
| I33d00 | | 20056910 | 13244110 | 6812800 | 66.03% | | 33.97% |
| I33d07 | | 27606518 | 11580054 | 16026464 | 41.95% | | 58.05% |
| I33d14 | | 27276292 | 10229420 | 17046872 | 37.50% | | 62.50% |
| I33d28 | | 25443474 | 15315588 | 10127886 | 60.19% | | 39.81% |
| I33d56 | | 27823884 | 15290090 | 12533794 | 54.95% | | 45.05% |
| I37d00 | | 27783280 | 23530652 | 4252628 | 84.69% | | 15.31% |
| I37d07 | | 23484788 | 11392218 | 12092570 | 48.51% | | 51.49% |
| I37d14 | | 24540726 | 12364340 | 12176386 | 50.38% | | 49.62% |
| I37d28 | | 30416030 | 16160890 | 14255140 | 53.13% | | 46.87% |
| I37d56 | | 25435626 | 15901300 | 9534326 | 62.52% | | 37.48% |
| I62d00 | | 25163510 | 12950888 | 12212622 | 51.47% | | 48.53% |
| I62d07 | | 30166002 | 28011488 | 2154514 | 92.86% | | 7.14% |
| I62d14 | | 26151448 | 11832710 | 14318738 | 45.25% | | 54.75% |
| I62d28 | | 25574892 | 14211802 | 11363090 | 55.57% | | 44.43% |
| I62d56 | | 26662906 | 13422722 | 13240184 | 50.34% | | 49.66% |
| I63d00 | | 25731580 | 15372190 | 10359390 | 59.74% | | 40.26% |
| I63d07 | | 23328014 | 9260740 | 14067274 | 39.70% | | 60.30% |
| I63d14 | | 23768796 | 19148346 | 4620450 | 80.56% | | 19.44% |
| I63d28 | | 26252494 | 11482078 | 14770416 | 43.74% | | 56.26% |
| I63d56 | | 29248516 | 13995874 | 15252642 | 47.85% | | 52.15% |
| I65d00 | | 16115694 | 15988060 | 127634 | 99.21% | | 0.79% |
| I65d07 | | 24065096 | 15095078 | 8970018 | 62.73% | | 37.27% |
| I65d14 | | 22594116 | 9630478 | 12963638 | 42.62% | | 57.38% |
| I65d28 | | 27326870 | 13216602 | 14110268 | 48.36% | | 51.64% |
| I65d56 | | 26392800 | 14214008 | 12178792 | 53.86% | | 46.14% |
| P01d00 | | 20918728 | 20598034 | 320694 | 98.47% | | 1.53% |
| P01d07 | | 15840100 | 9699378 | 6140722 | 61.23% | | 38.77% |
| P01d14 | | 20651164 | 12586454 | 8064710 | 60.95% | | 39.05% |
| P01d28 | | 21849230 | 12491422 | 9357808 | 57.17% | | 42.83% |
| P01d56 | | 22414040 | 12798762 | 9615278 | 57.10% | | 42.90% |
| P02d00 | | 20773112 | 17866194 | 2906918 | 86.01% | | 13.99% |
| P02d07 | | 19439288 | 10789860 | 8649428 | 55.51% | | 44.49% |
| P02d14 | | 23434722 | 19597482 | 3837240 | 83.63% | | 16.37% |
| P02d28 | | 23186620 | 12344584 | 10842036 | 53.24% | | 46.76% |
| P02d56 | | 19207070 | 11637040 | 7570030 | 60.59% | | 39.41% |
| P05d00 | | 13976614 | 1562504 | 12414110 | 11.18% | | 88.82% |
| P05d07 | | 18847616 | 11566910 | 7280706 | 61.37% | | 38.63% |
| P05d14 | | 23906778 | 13009538 | 10897240 | 54.42% | | 45.58% |
| P05d28 | | 22003704 | 13339798 | 8663906 | 60.63% | | 39.37% |
| P05d56 | | 20807192 | 13231076 | 7576116 | 63.59% | | 36.41% |
| P12d00 | | 24325942 | 15786090 | 8539852 | 64.89% | | 35.11% |
| P12d07 | | 20182760 | 15330778 | 4851982 | 75.96% | | 24.04% |
| P12d14 | | 16642110 | 9432396 | 7209714 | 56.68% | | 43.32% |
| P12d28 | | 21540914 | 11530868 | 10010046 | 53.53% | | 46.47% |
| P12d56 | | 23011764 | 12791394 | 10220370 | 55.59% | | 44.41% |
| P32d00 | | 18143628 | 17360760 | 782868 | 95.69% | | 4.31% |
| P32d07 | | 17782522 | 14683662 | 3098860 | 82.57% | | 17.43% |
| P32d14 | | 20421972 | 14214748 | 6207224 | 69.61% | | 30.39% |
| P32d28 | | 24837064 | 14481408 | 10355656 | 58.31% | | 41.69% |
| P32d56 | | 20448612 | 12601448 | 7847164 | 61.62% | | 38.38% |
| P39d00 | | 22127320 | 12488076 | 9639244 | 56.44% | | 43.56% |
| P39d07 | | 31527016 | 11672174 | 19854842 | 37.02% | | 62.98% |
| P39d14 | | 20128638 | 10177662 | 9950976 | 50.56% | | 49.44% |
| P39d28 | | 21220648 | 10613544 | 10607104 | 50.02% | | 49.98% |
| P39d56 | | 26131856 | 12729082 | 13402774 | 48.71% | | 51.29% |
| P42d00 | | 13708128 | 10831270 | 2876858 | 79.01% | | 20.99% |
| P42d07 | | 12892574 | 6441816 | 6450758 | 49.97% | | 50.03% |
| P42d14 | | 19627060 | 11411832 | 8215228 | 58.14% | | 41.86% |
| P42d28 | | 29810820 | 15396418 | 14414402 | 51.65% | | 48.35% |
| P42d56 | | 26662380 | 12525796 | 14136584 | 46.98% | | 53.02% |
| P45d00 | | 13870512 | 13785020 | 85492 | 99.38% | | 0.62% |
| P45d07 | | 18396844 | 9029462 | 9367382 | 49.08% | | 50.92% |
| P45d14 | | 23479684 | 9185494 | 14294190 | 39.12% | | 60.88% |
| P45d28 | | 22476172 | 13285314 | 9190858 | 59.11% | | 40.89% |
| P45d56 | | 26216964 | 13081334 | 13135630 | 49.90% | | 50.10% |
| P52d00 | | 20022136 | 10954394 | 9067742 | 54.71% | | 45.29% |
| P52d07 | | 14111230 | 4241750 | 9869480 | 30.06% | | 69.94% |
| P52d14 | | 22891210 | 10870088 | 12021122 | 47.49% | | 52.51% |
| P52d28 | | 18525968 | 12029992 | 6495976 | 64.94% | | 35.06% |
| P52d56 | | 27509348 | 12002462 | 15506886 | 43.63% | | 56.37% |
| SUM | 2779248798 | | 1586617206 | 1192631592 | |  |  |
| AVE | | 22780728 | 13005059 | 9775669 | 58.17% | | 41.83% |

1. **Supplementary Figures and Tables**


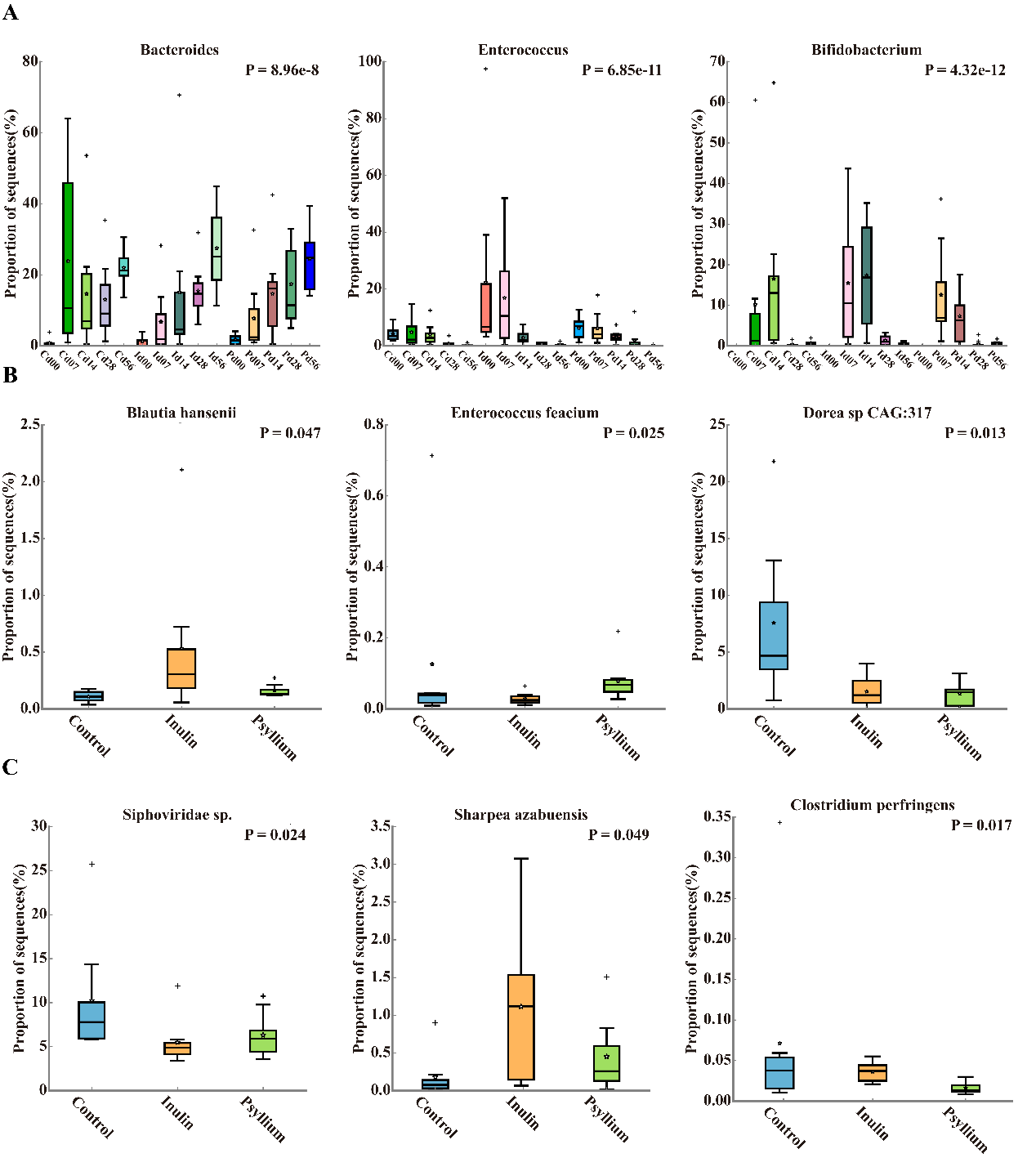


**Supplementary Figure 1.** Depicts the relative abundance of intestinal microbial taxa at the genus and species levels in calves with diarrhea fed dietary fiber supplementation, represented by box plots. (A) Box plots illustrating the relative abundance of genera such as Bacteroides, Enterococcus, and Bifidobacterium in the Con, Inu, and PHP groups at different time points.(B) Box plots showing the relative abundance of Blautia hansenii, Enterococcus faecium, and Dorea sp. CAG: 317 in the intestines of 28-day-old calves fed different dietary fibers.(C) Box plots representing the relative abundance of Siphoviridae sp., Sharpea azabuensis, and Clostridium perfringens in the intestines of 56-day-old calves fed different dietary fibers.Intergroup differences in (A), (B), and (C) were assessed using the Kruskal-Wallis test, with P < 0.05 indicating significant differences and P < 0.01 indicating highly significant differences.


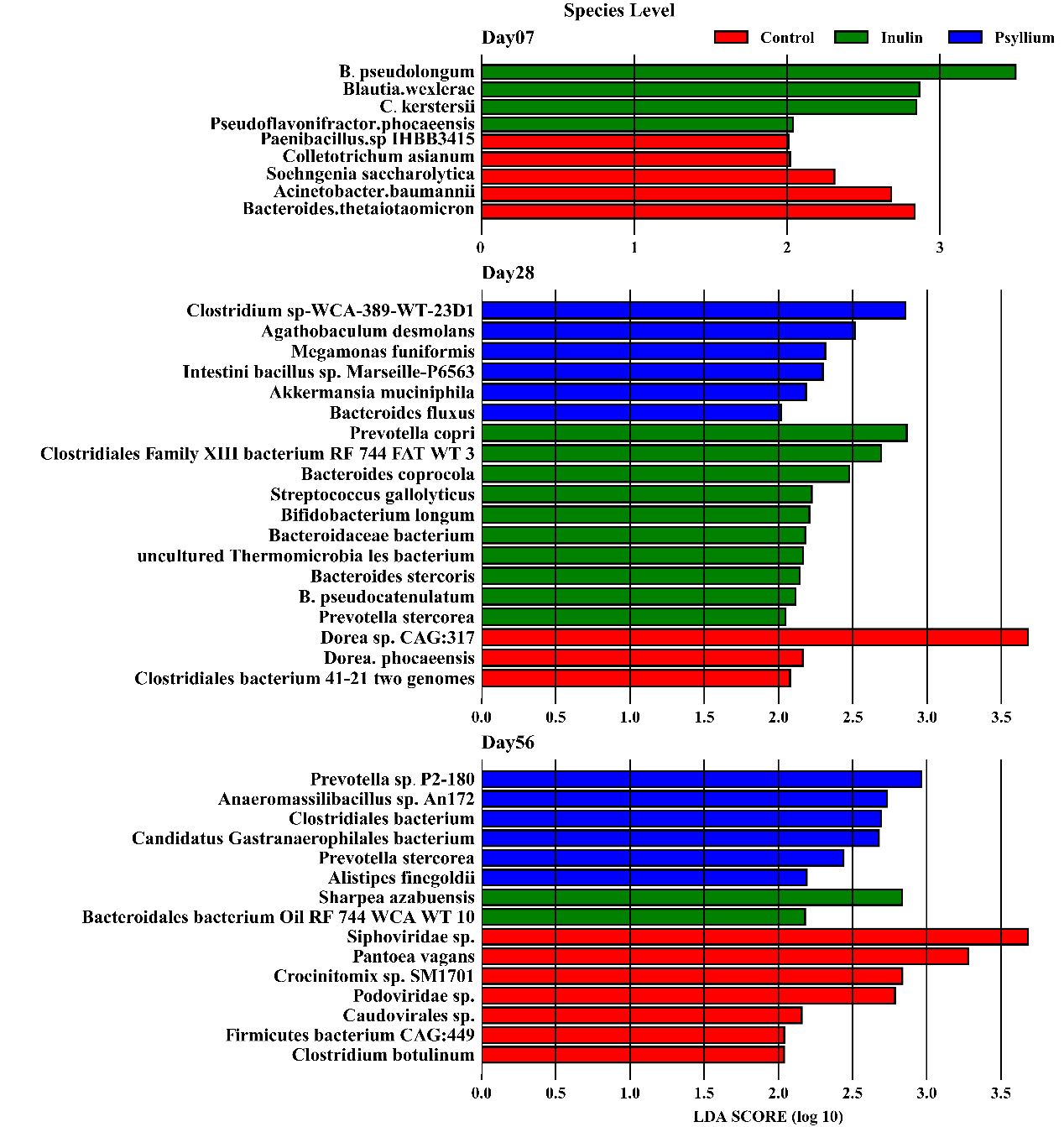


**Supplementary Figure 2.** LEfSe analysis shows the microbiota compositions at the species level between three groups (P < 0.05 and LDA > 2).


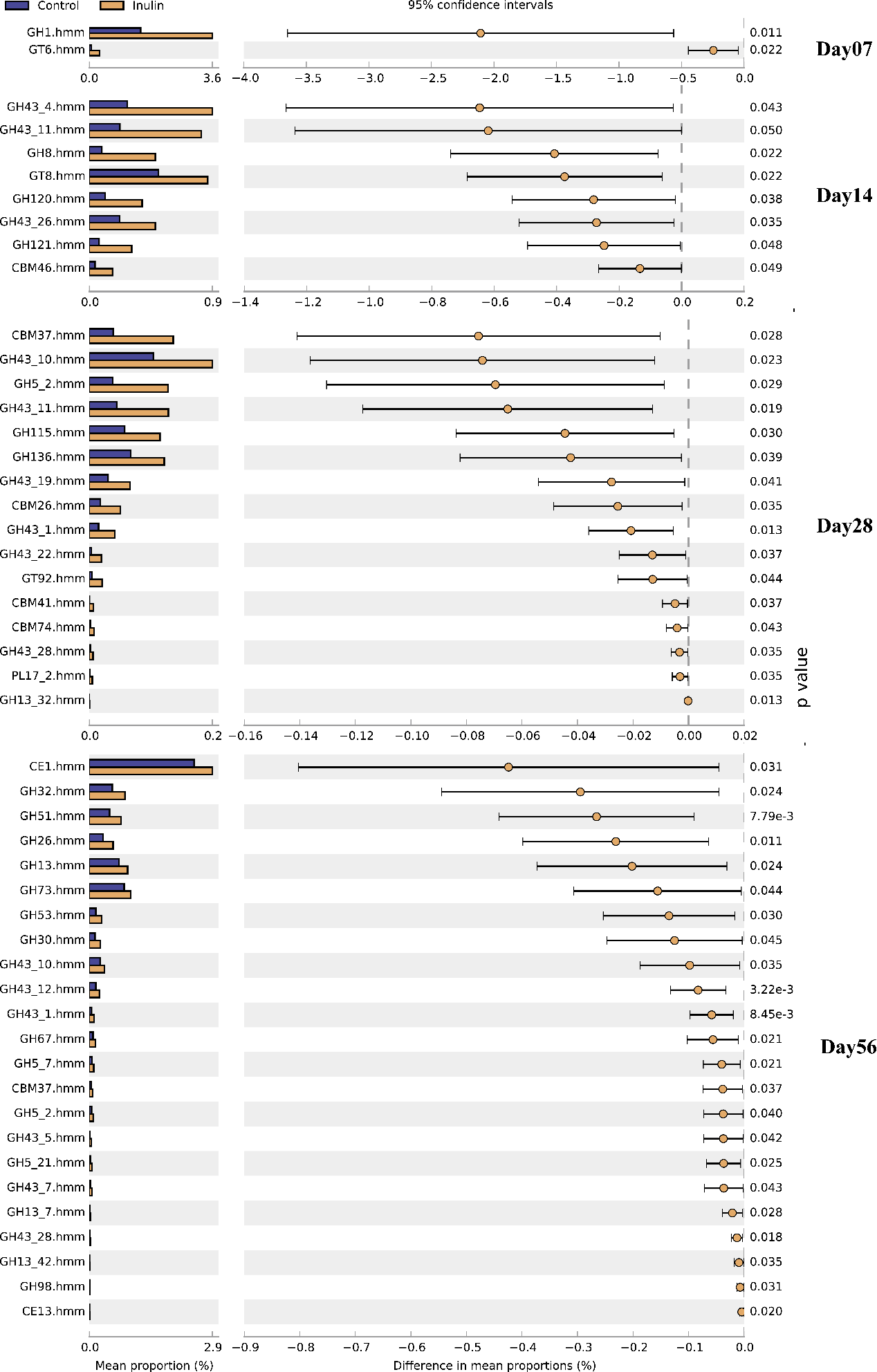


**Supplementary Figure 3.** The Statistical Analysis of Taxonomic and Functional Profiles (STAMP) was employed to assess the differences in the relative abundance of Carbohydrate-Active Enzymes (CAZy) between the Con and Inu groups. Specifically, the relative abundance of CAZy in calves supplemented with inulin was compared to that of the Con group at 7, 14, 28, and 56 days of age to determine differences. The statistical test employed was the two-sided Welch's t-test, and the confidence interval method used was DP: Welch's inverted 0.95. Differences were considered significant if P < 0.05 and highly significant if P < 0.01.


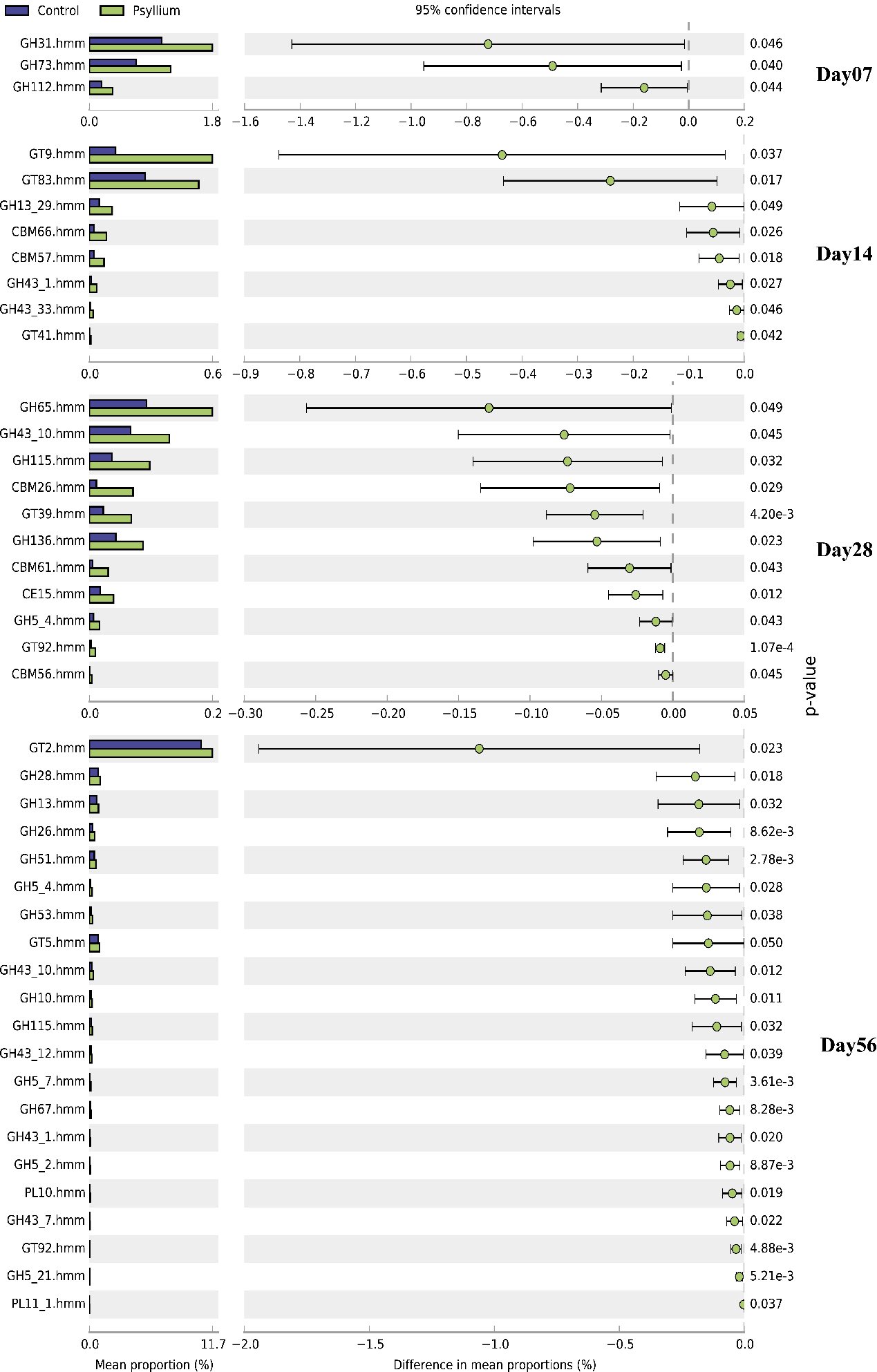


**Supplementary Figure 4.** The Statistical Analysis of Taxonomic and Functional Profiles (STAMP) was utilized to assess the differences in the relative abundance of Carbohydrate-Active Enzymes (CAZy) between the Con and PHP groups. Specifically, the relative abundance of CAZy in calves supplemented with Psyllium husk was compared to that of the Con group at 7, 14, 28, and 56 days of age to determine differences. The statistical test employed was the two-sided Welch's t-test, and the confidence interval method used was DP: Welch's inverted 0.95. Differences were considered significant if P < 0.05 and highly significant if P < 0.01.
